# Allosteric Modulator Leads Hiding in Plain Site: Developing Peptide and Peptidomimetics as GPCR Allosteric Modulators

**DOI:** 10.3389/fchem.2021.671483

**Published:** 2021-10-07

**Authors:** Keith M. Olson, John R. Traynor, Andrew Alt

**Affiliations:** ^1^ Department of Pharmacology and Edward F Domino Research Center, University of Michigan, Ann Arbor, MI, United States; ^2^ Department of Medicinal Chemistry, College of Pharmacy, University of Michigan, Ann Arbor, MI, United States; ^3^ Life Sciences Institute, University of Michigan, Ann Arbor, MI, United States

**Keywords:** G-protein coupled receptor, pepducin, peptidomimetic, biased signaling, allosteric modulators, drug discovery, nanobodies, peptide

## Abstract

Allosteric modulators (AMs) of G-protein coupled receptors (GPCRs) are desirable drug targets because they can produce fewer on-target side effects, improved selectivity, and better biological specificity (e.g., biased signaling or probe dependence) than orthosteric drugs. An underappreciated source for identifying AM leads are peptides and proteins—many of which were evolutionarily selected as AMs—derived from endogenous protein-protein interactions (e.g., transducer/accessory proteins), intramolecular receptor contacts (e.g., pepducins or extracellular domains), endogenous peptides, and exogenous libraries (e.g., nanobodies or conotoxins). Peptides offer distinct advantages over small molecules, including high affinity, good tolerability, and good bioactivity, and specific disadvantages, including relatively poor metabolic stability and bioavailability. Peptidomimetics are molecules that combine the advantages of both peptides and small molecules by mimicking the peptide’s chemical features responsible for bioactivity while improving its druggability. This review 1) discusses sources and strategies to identify peptide/peptidomimetic AMs, 2) overviews strategies to convert a peptide lead into more drug-like “peptidomimetic,” and 3) critically analyzes the advantages, disadvantages, and future directions of peptidomimetic AMs. While small molecules will and should play a vital role in AM drug discovery, peptidomimetics can complement and even exceed the advantages of small molecules, depending on the target, site, lead, and associated factors.

## Introduction: Peptide and Peptidomimetics Are Appealing Sources for Allosteric Modulator Drug Discovery

**FIGURE 1:**
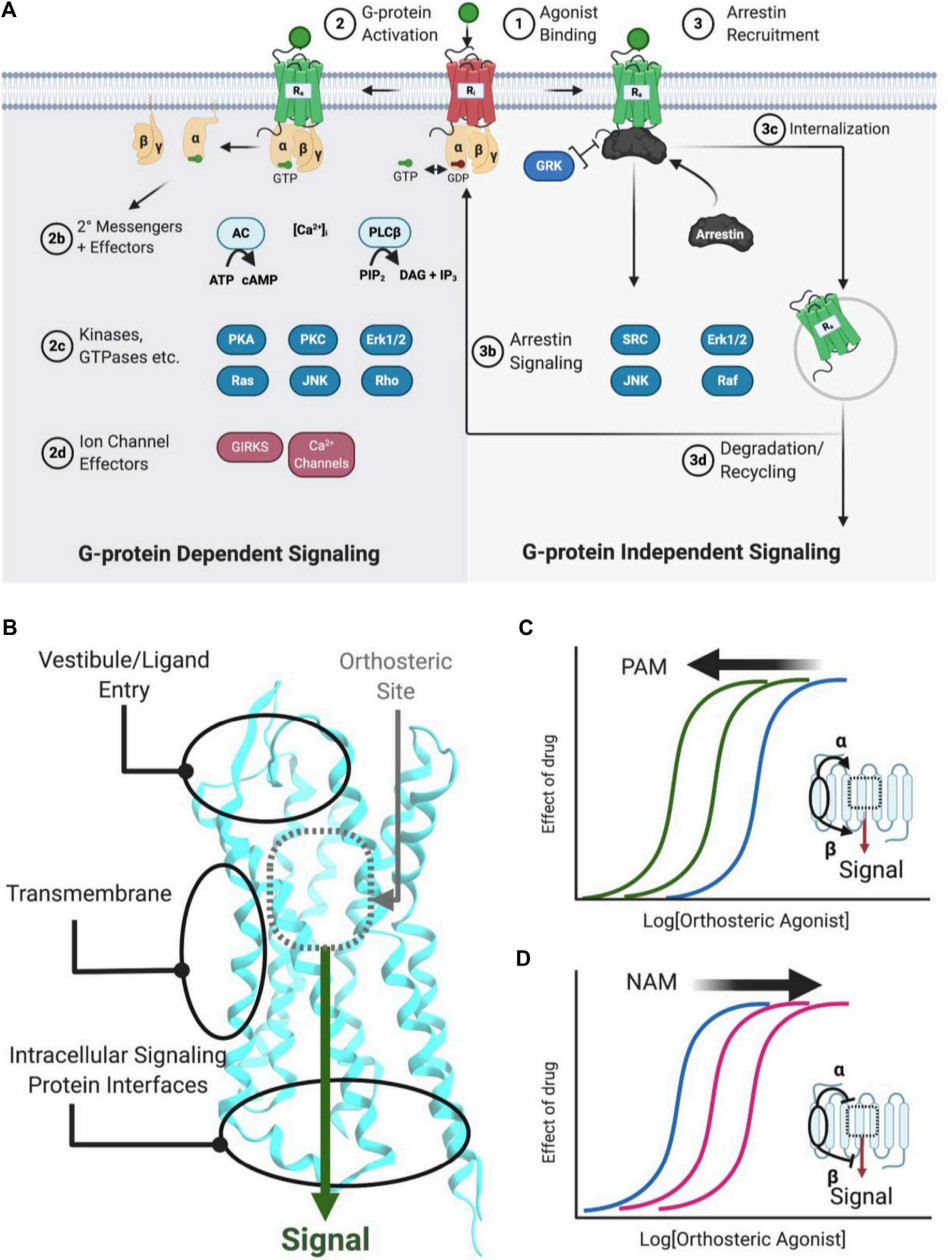
Simplified schematic of GPCR signaling pathways, AM binding sites, and positive/negative AMs. **(A)** Common GPCR signaling pathways are activated by an orthosteric agonist (green circle), which converts an inactive receptor (red, R_i_) to an active receptor (green, R_a_) (Step 1). R_a_ causes the transducer G-protein to exchange GDP for GTP and modulate various downstream effectors, such as kinases, ion channels, and enzymes (Step 2). Classically, signaling is terminated by phosphorylation, arrestin recruitment, and internalization (Step 3). Furthermore, arrestins can scaffold signaling pathways. Biased agonists can favor specific pathways, such as **(right)** preferentially stimulating arrestin signaling over **(left)** G-protein dependent signaling, or vice versa. **(B)** Classical agonists and antagonists bind to the orthosteric site (gray dotted box), as shown for Class A GPCRs. GPCR crystal structures with AMs show three general binding sites, the vestibule and ECLs in the extracellular matrix, the transmembrane region, which contacts the lipid membrane. Several different sites may exist in any of these general categories. **(C)** PAMs stabilize a conformation that increases orthosteric agonist affinity (α), as shown by the curve shift. Additionally, PAMs can increase the signaling efficacy (β) of orthosteric agonists (not shown). **(D)** NAMs decrease the potency of orthosteric agonists (as shown) by stabilizing a conformation that reduces affinity (α) or signaling efficacy (β). Figure was created with BioRender.com ^1^ and Molecular Operating Environment (MOE).^2^ PDB ID: 5C1M.A (Huang et al., 2015).

**FIGURE 2:**
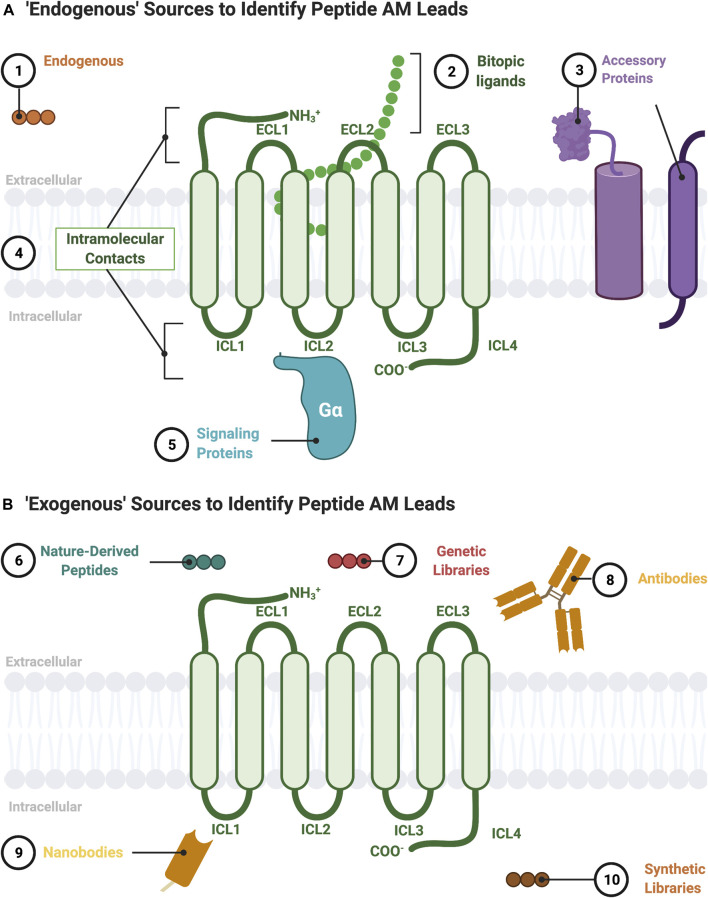
Sources to identify peptide AM leads. **(A)** “Endogenous” sources of peptide AMs include the following: 1) endogenous peptide sequences such as orthosteric ligands from other receptors or protein fragments; 2) nonorthosteric site residues from large endogenous bitopic peptides or hormones; 3) accessory protein protein-protein interactions, such as RAMPs, heterooligomers, and RTKs; 4) intramolecular contacts, such as sequences from the ICL (pepducins) or ECL regions; 5) signaling protein protein-protein interactions, such as Gα. **(B)** “Exogenous” sources for peptide AMs include 6) nature-derived peptides, such as from natural product libraries, conotoxins, cyclotides, and snake/scorpion venom; 7) genetic libraries, such as phage-display or directed evolution platforms; 8) antibodies including engineered and autoantibodies; 9) nanobodies; 10) synthetic libraries, such as combinatorial or DNA-encoded libraries. Figure was created with BioRender.com ^1^.

G-protein coupled receptors are transmembrane signaling proteins targeted by an estimated ∼35% of clinically approved drugs that usually bind to the same conserved “orthosteric site” as the endogenous agonist (Sriram and Insel, 2018) (Figure 1). Additionally, GPCRs possess discrete "allosteric sites’ whereby ligands, including endogenous peptides and proteins, can modulate the activity of the orthosteric agonist(s) (Figures 1C,D; Table 1). Allosteric modulators (AMs) are desirable drug targets because they can fine-tune receptor activity while retaining the spatial and temporal signaling profile of endogenous ligands leading to drugs with fewer on-target side effects, improved subtype selectivity, and perhaps facilitate biased signaling better than orthosteric ligands (Kenakin and Christopoulos, 2013; Wootten et al., 2013). Peptides and proteins—many of which were evolutionarily selected to modulate GPCR signaling—are underappreciated as AM leads with potential sources including endogenous protein-protein interactions (e.g., transducer or accessory proteins), intramolecular receptor contacts (e.g., pepducins or extracellular domains), endogenous peptide modulators (e.g., protein fragments or “endogenous bitopic” ligands), and exogenous sources (e.g., nanobodies, conotoxins, synthetic peptide libraries, or nature-derived peptides) (Figure 2).

**TABLE 1:** Key definitions and terms.

Term	Definition
ago-PAMs	Positive allosteric modulator agonists (ago-PAMs) stabilize a receptor’s active conformation in the absence of an orthosteric agonist.
AMs	Allosteric modulators (AMs) bind to a spatially distinct site capable of fine-tuning orthosteric ligand activity.
Allosteric site	A spatially distinct binding site, which does not bind the endogenous orthosteric agonist.
Amino acid scans	Systematic conversion of peptide residues, one by one, to determine structural and physical features of pharmacophore.
Biased AMs	Biased allosteric modulators (biased-AMs) favor activation of one set of pathways over another set activated by an orthosteric agonist or as ago-PAMs.
Bioavailability	The extent a drug can reach its intended target, including membrane-, gut-, or blood-brain barrier permeability.
Bioisostere	A chemical substitution with similar electronic and physical properties.
Bitopic ligand	A ligand that binds to both the orthosteric and an allosteric site.
Conjugation	Addition of a moiety to a molecule to induce a specific property, such as improved bioavailability or solubility.
ECL	The extracellular loops (ECLs) of the GPCR that contain the vestibule.
Global modifications	Peptidomimetic strategy covalently binding two non-sequential peptides residues.
ICL	The intracellular loops (ICLs) of the GPCR connecting TMs.
Local modification	A modification of 1 or 2 sequential residues (e.g., amide or sidechain bioisosteres).
NALs	Neutral allosteric ligands (NALs) bind an allosteric site without changing orthosteric ligand activity.
NAMs	AMs that decrease orthosteric agonist activity by altering affinity and/or efficacy.
Orthosteric ligand	A ligand that binds to the same site of the endogenous agonists.
Orthosteric Site	A generally conserved site to which endogenous agonists and most classical GPCR drugs bind.
PAMs	AMs which enhance orthosteric agonist affinity and/or efficacy.
Pepducin	A peptide AM derived from a receptor’s ICLs attached to a lipid moiety, which typically stabilizes a receptor state by binding to the intracellular face.
Peptide	A molecule containing two or more amide bonds.
Peptidomimetics	Peptide-like molecules with modifications to improve drug-like properties and/or bioactivity.
Pharmacophore	The key chemical, H-bond donors/acceptors, aromatic/hydrophobic groups, charges, and so om, and structural features required for bioactivity.
Protein-protein interactions	The interface between two proteins or protein peptide, which a drug could inhibit.
Probe-dependent AMs	Probe-dependent allosteric modulators shift the potency/efficacy of one ligand but not another.
SAR	Structure-activity relationship (SAR) studies determine the effect of altering a ligand’s chemical and structural features on bioactivity, metabolic stability, or related properties.
Secondary structure	The 3D arrangement of a linear peptide sequence stabilized by amide H-bond donors and carbonyl C=O acceptors, such as α-helices, β-sheets, or β-loops.
TMs	The transmembrane helical domains (TMs) of the GPCR.
Truncations/deletions	The systematic removal or deletion of peptide residues one by one.

**FIGURE 3:**
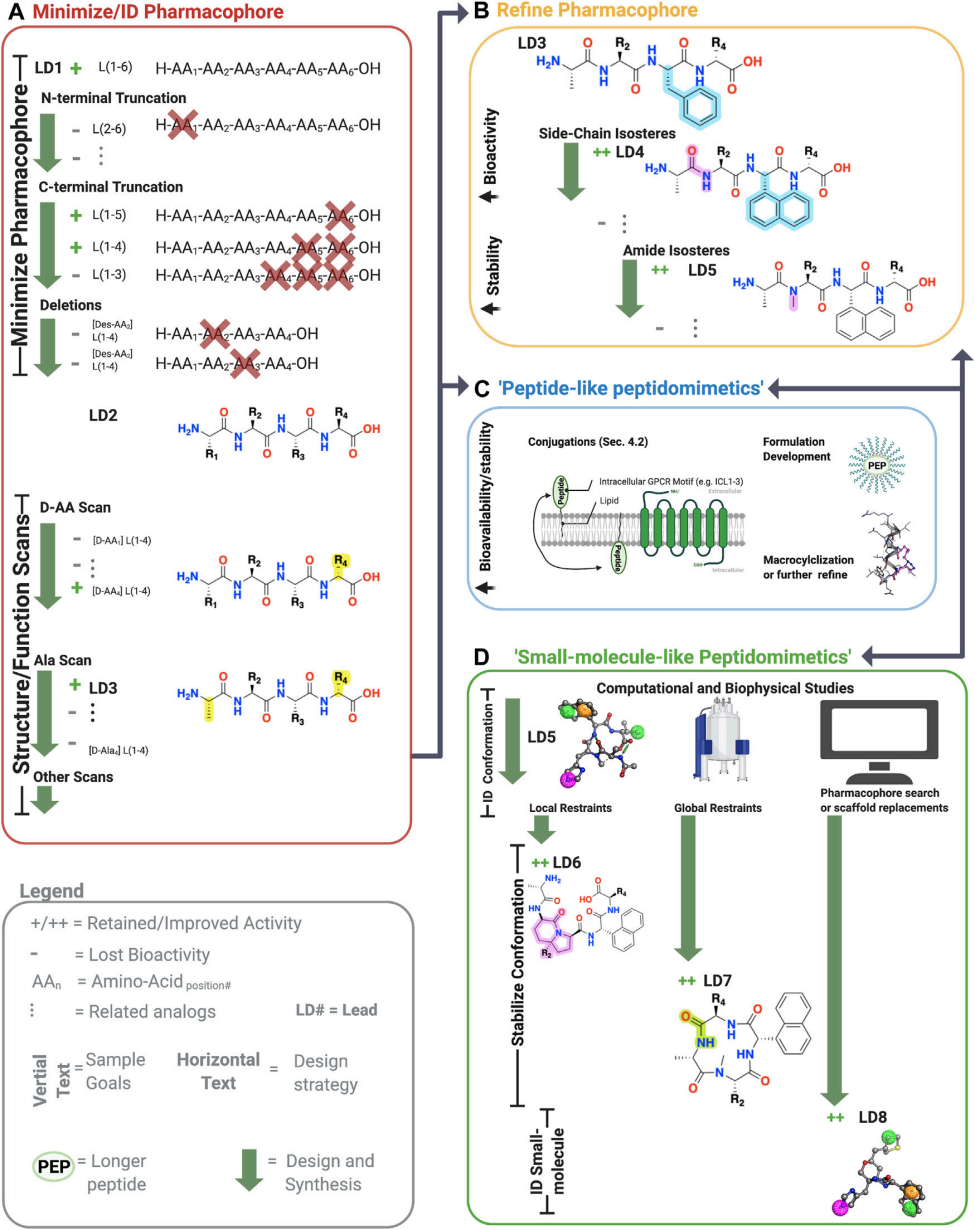
Sample workflow for peptidomimetic drug development. Sample workflow for peptidomimetic drug design of a hypothetical six-amino acid peptide lead LD1[L(1-6)]. **(A)** First, pharmacophore minimization occurs involving truncations of N-terminus/C-terminus and a deletion scan. In this case, the N-terminal truncation of AA_1_ abolished bioactivity (gray), and thus L(1-6) was retained. The C-terminal removal of AA_5_ and AA_6_ retained activity but not AA_4_ yielding L(1-4) or LD2. Next, deletion of the remaining amino acids is tested to further reduce the lead’s molecular weight. In the example, deleting either AA_2_ or AA_3_ abolished activity, leaving the lead unchanged L(1-4). Finally, sequential amino acid scans can identify the conformational and side-chain requirements for the pharmacophore. In the example, AA_1_→alanine **(A)** and L*-AA_4_ → D*-AA_4_ retained activity, yielded [ala_1_, D-AA_4_] L(1-4), referred to LD3. At this point, one can perform additional scans or progress to step B, C, or D. **(B)** The pharmacophore and bioactivity can be further refined depending on the goals. In our example, a side-chain isostere of the F_3_ improved bioactivity, while amide N-methylation improved metabolic stability, yielding LD4 and LD5, respectively. In principle, any modification can alter bioactivity or stability. **(C)** Peptide-like peptidomimetics of longer or less stable sequences can be optimized through various conjugations, such as lipidation to improve membrane permeability, macrocyclizations to stabilize the conformation and improve metabolic stability, or formulation development to make large peptides orally available. For example, lipid conjugations (black squiggly line) can improve membrane permeability of long peptides (green oval) by facilitating insertion into the membrane, followed by ‘membrane flipping’ to the intracellular surface. **(D)** 3-dimensionally compact pharmacophores or shorter sequences can undergo ‘small-molecule-’ like peptidomimetic development to improve desired bioactivity and druggability by stabilizing the bioactive conformation via global and local modifications, pharmacophore searches of small molecule libraries, scaffold mimetics of peptide secondary structures, or scaffold replacements using the biophysical analyses (e.g., NMR or crystallography) and computational studies (e.g., docking, conformation predictions, QSAR). Once bioactive conformation is identified, SARs iteratively (two-headed arrows) investigate the changes in activity based on structural modifications using computational or biophysical characterization. +, retained activity; ++, improved activity;−, lost activity. * indicates chirality and not a single letter amino acid. Figure was created with BioRender.com ^1^ and Molecular Operating Environment (MOE).^2^

Advances in peptide chemistry and drug formulations have increased peptide’s share in the drug market, with more than 150 peptide/peptidomimetic drugs in clinical development and at least 68 approved for human use (Lau and Dunn, 2018). From 2011 to 2018, the market share for peptide therapeutics grew rapidly, from $14.1 billion to $24.5 billion (Lau and Dunn, 2018). The benefits of peptide leads include high affinity, good bioactivity, good solubility, and high tolerability compared to small molecules, especially early on in drug discovery [e.g., Hruby et al. (2017)]. While peptide scaffolds do have disadvantages, including poor metabolic stability and low bioavailability (Lenci and Trabocchi, 2020), these classical barriers are increasingly manageable through “peptidomimetic” design. Here, we define peptidomimetics broadly as molecules that combine the advantages of both peptides and small molecules by mimicking the peptide’s chemical features responsible for bioactivity while improving its bioavailability and metabolic stability (Figure 3). As such, peptide/peptidomimetics can complement and even exceed the advantages of small molecule drug discovery, depending on the target, disease, and related considerations (Henninot et al., 2018).

**FIGURE 4:**
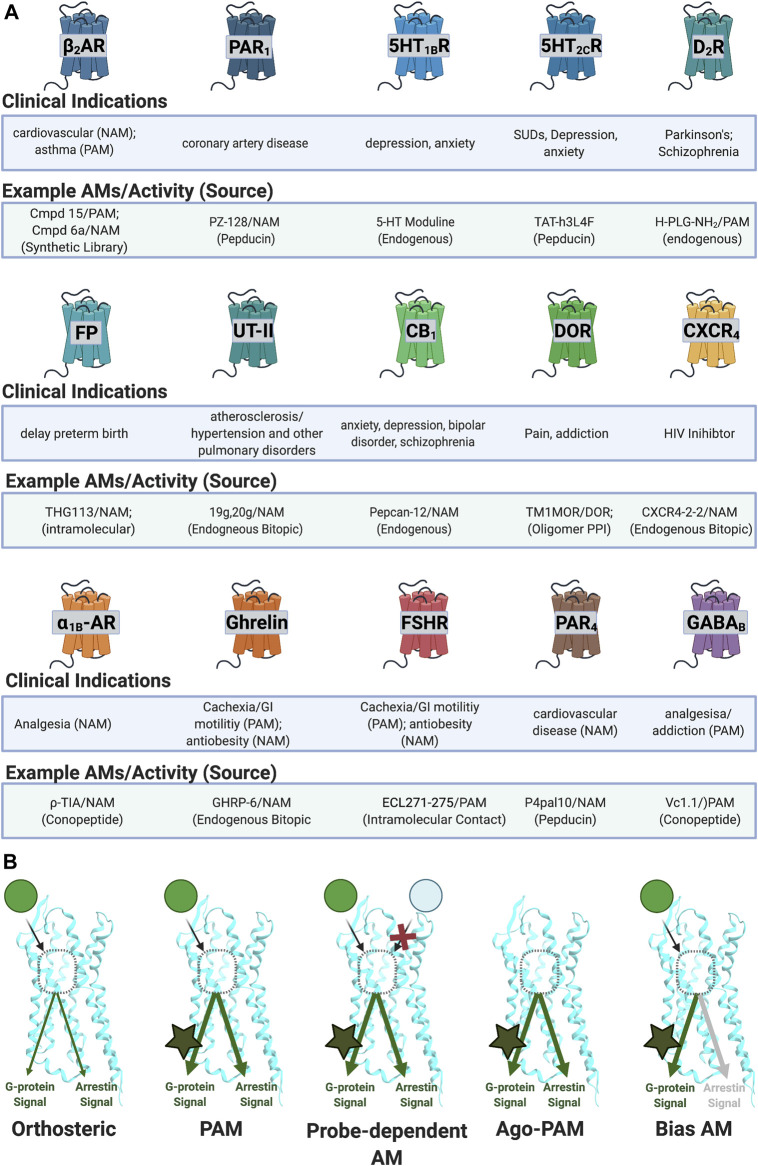
Reported AM targets for peptide/peptidomimetics therapeutics. **(A)** Sample of clinical indications of peptidomimetics discussed in this review. **(B)** AMs can produce various biological profiles. **(Left)** PAMs potentiate signals regardless of ligand or downstream mechanism. **(Middle left)** probe-dependent AMs potentiate the effects of one agonist (e.g., green) but not another agonist (e.g., light blue). **(Middle right)** ago-PAMs activate the receptor in the absence of an orthosteric ligand. **(Right)** bias-AMs preferentially activate one set of signaling pathways over another, either in the presence of an orthosteric agonist or as an ago-PAM, including but not limited to Gα versus β-arrestin signaling. Figure was created with BioRender.com.^1^

**FIGURE 5:**
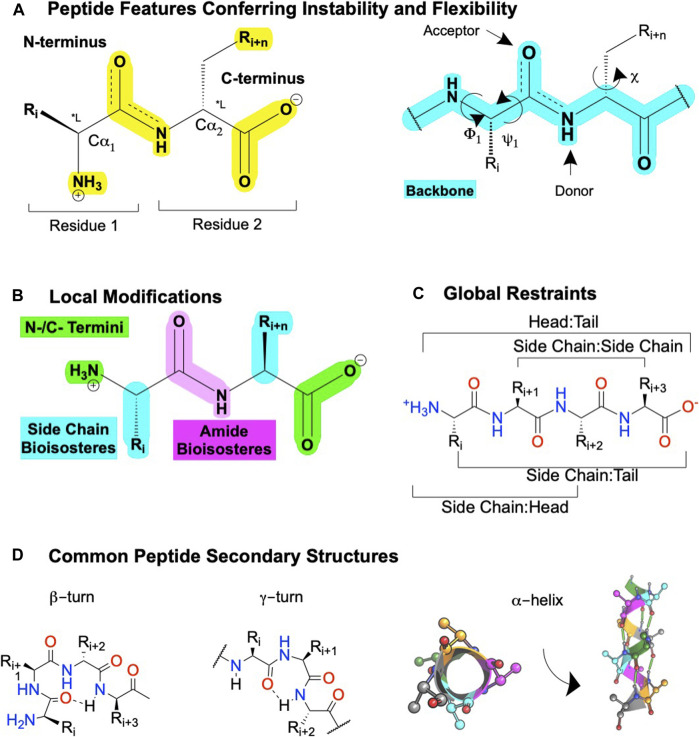
Peptide features and peptidomimetic modifications to improve their drug-like properties. **(A)** Peptide nomenclature dictates sequences are written left to right starting with the N-terminal labeled as "residue 1′, with each residue connected by an amide bond, i + n **(left)**. Sites for local modifications are highlighted in yellow **(right)**, with the peptide backbone highlighted in cyan. The peptide nomenclature and types of peptidomimetic modifications to improve drug-like properties of linear peptides include **(B)** local substitutions of side chains (cyan), modifications of the C- and N- termini (green), and amide bioisosteres (pink). **(C)** Global modifications. **(D)** Common secondary structures for peptides including at β-turn, a γ-turn, and **(left)** a α-helix top-down view or **(right)** α-helix side view. Dotted lines or green cylinders show backbone H-bonds. Figure was created with BioRender.com,^1^ Chemdraw20.0, MarvinSketch,^5^ and Molecular Operating Environment (MOE).^2^

**FIGURE 6:**
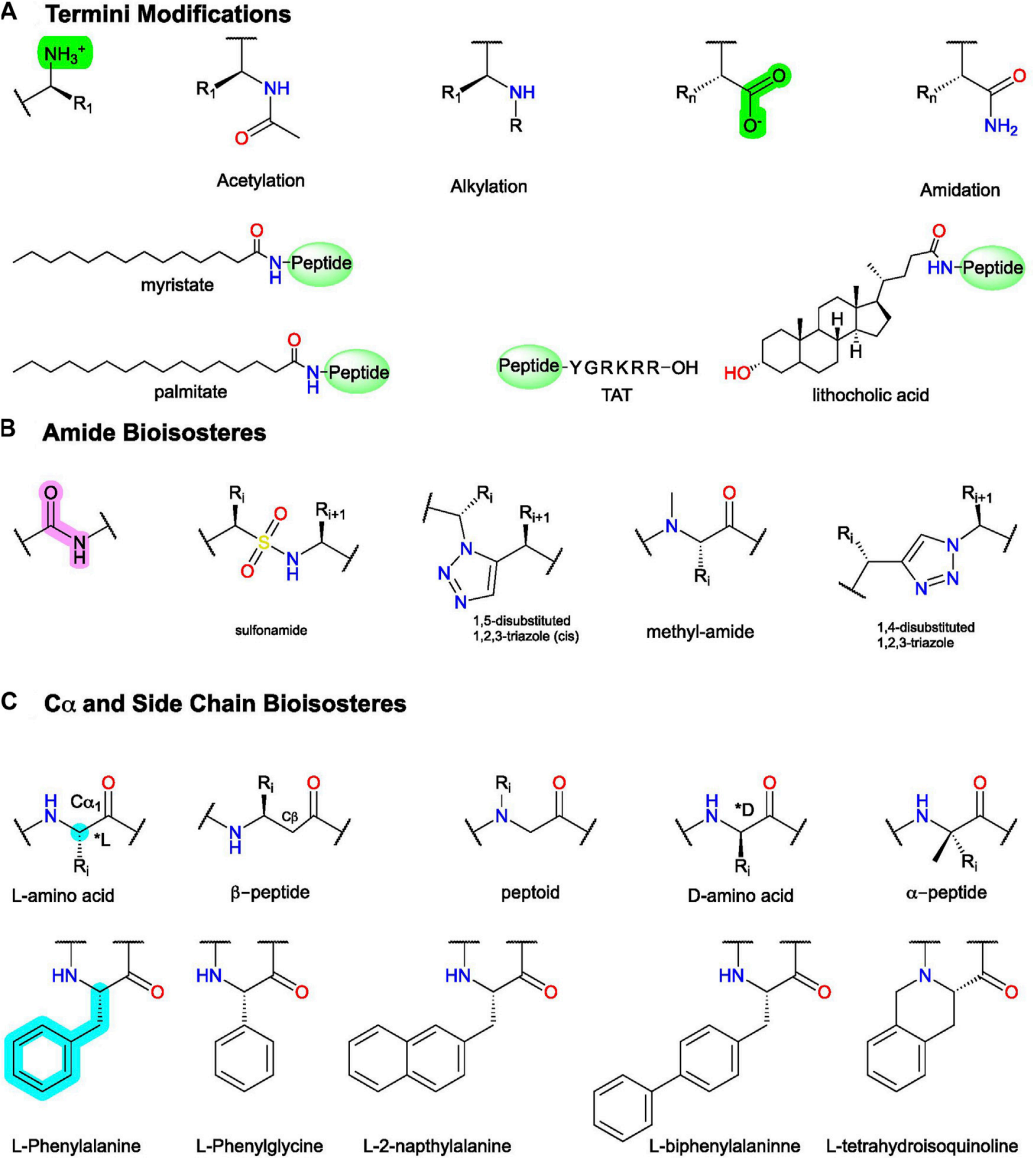
Example peptidomimetic modifications to improve activity, metabolic stability, or bioavailability of peptides. **(A)** Conjugation and C- and N-termini modifications generally improve peptides’ metabolic stability and bioavailability. **(B)** Amide bioisosteres replicate the electronic and physiochemical properties of the amide bond (pink). **(C)** **(Top)** example modifications to Cα and **(bottom)** sidechain isosteres of phenylalanine (F). Figure was created with Chemdraw20.0.

In fact, several peptide/peptidomimetic AMs have entered clinical trials, including PZ-128 at the protease-activated receptor 1 (PAR1) (Kuliopulos et al., 2020) and Vc1.1 at Gamma-aminobutyric acid B receptors (GABA_B_),^3,^ ^4^ highlighting their ability to achieve clinically acceptable drug-like properties. Moreover, the 31-amino acid Semaglutide (MW = 4,114)—marketed as Rybelsus®—is an orally available FDA-approved drug to treat type 2 diabetes (FDA, 2020). With the clinical success of large orally available peptides, the appeal of AMs as drug targets, and the observation that peptides/proteins act as endogenous AMs, a review focusing on peptide/peptidomimetic AMs is warranted. Several excellent reviews exist covering small molecule AMs [e.g., Lu and Zhang (2019), Wold et al. (2019)]; however, there is no comprehensive review of peptide/peptidomimetic AMs, despite numerous examples in the literature (Figures 3, 4). Therefore, this review provides the following:

1) An overview of AM profiles and advantages over orthosteric ligands
2) Sources to identify AM peptide/peptidomimetic leads (Figure 2)
3) Strategies to convert a peptide lead into more drug-like “peptidomimetic” (Figures 3, 5, 6)
4) Limitations and future directions of peptide/peptidomimetic AMs

## GPCR Allosteric Modulators Refine Orthosteric Ligand Activity

### Endogenous Orthosteric Agonists Directly Activate Transducers, Such as the Heterotrimeric Gαβγ Proteins, Leading to Downstream Signal Transduction

GPCRs are highly flexible and dynamic receptors that adopt many different conformations or ensembles. Classically, orthosteric agonist binding converts the inactive receptor conformation(s) (R_I_) into the active receptor conformation(s) (R_A_) (Figure 1A; Step 1), causing the Gα transducer to exchange GDP for GTP, followed by disassociation of the heteromeric Gαβγ (Kenakin and Christopoulos, 2013) (Figure 1A; Step 2). Depending on the receptor, various Gα and Gβγ subtypes modulate downstream effectors, including kinases, ion channels, enzymes, and their secondary messengers (Figure 1A; Steps 2b−d). In total, 16 Gα subunits (McCudden et al., 2005), six Gβ subunits, and twelve Gγ subtypes have been discovered (Khan et al., 2013) that show different preferences activating (or inactivating) specific signaling effectors/pathways. For example, receptors coupled to Gα_s_ activate adenylyl cyclase (AC), leading to increased cAMP concentrations, whereas receptors coupled to Gα_i/o_ inhibit AC cAMP production. Next, β-arrestins traditionally terminate G-protein signaling by desensitizing the R_A_ and mediating clathrin-dependent internalization (Figure 1A; step 3c) and/or act as a scaffold for effectors, such as Src, ERK1/2, JNK, and Raf (Figure 1A; Step 3b) (Kenakin and Christopoulos, 2013). A generic simplified GPCR signaling schematic is shown in Figure 1A, though it is important to note the specifics vary between different receptors, ligands, cell types, and signaling proteins.

AMs increase or decrease an orthosteric ligand’s efficacy—the degree of the biological response produced in a cellular or physiological readout—and potency—the ligand concentration that produces a half-maximal effect—by binding to a spatially distinct site on the protein (Figure 1B). Based on current GPCR crystal structures, AMs drugs bind to three different receptor regions:

1) The extracellular surface [e.g., “vestibule” and extracellular loops (ECLs)]
2) The transmembrane (TM) helices
3) The intracellular face or intracellular loops (ICLs)

AMs typically shift orthosteric ligand potency and/or efficacy by stabilizing “molecular switches” required for converting the inactive conformation (R_i_) to the active conformation (R_a_) or altering the orthosteric ligand affinity by binding to the vestibule site (Lu and Zhang, 2019). Critically for drug development, allosteric sites are less evolutionarily conserved than orthosteric sites because of less selection pressure to recognize the endogenous orthosteric ligand(s). Consequently, allosteric sites can provide distinct topological and physicochemical features to exploit in drug development that enable better subtype selectivity and increased biological specificity (i.e., biased signaling or probe dependence) (Kenakin and Christopoulos, 2013; Wootten et al., 2013; Slosky et al., 2021), which are particularly desirable features in GPCR drug development.

### Positive Allosteric Modulators

PAMs increase orthosteric agonist affinity (α) and/or efficacy (β) without directly activating the receptor. In principle, this retains the endogenous agonists’ temporal and spatial specificity by only enhancing signals in tissues exposed to endogenous agonists (Burford et al., 2015). As such, PAMs can act as “molecular amplifiers” of endogenous (or exogenous) orthosteric agonists, augmenting a submaximal signal (Figure 1C). In compartments or tissues where the endogenous agonist is not present, a PAM remains “silent” even when it is bound to the receptor (Burford et al., 2011). By this mechanism, PAMs can reduce “on-target side effects” compared to exogenous orthosteric agonists by avoiding untoward effects that result from the indiscriminate receptor activation produced by exogenous agonists (Burford et al., 2015; Lu and Zhang, 2019).

### Negative Allosteric Modulators

NAMs decrease orthosteric agonist signaling by reducing its affinity (α) and/or efficacy (β) and often increase the affinity of orthosteric antagonists’ (Figure 1D). NAMs provide several advantages over orthosteric antagonists, including partial—but not complete—reduction of endogenous signaling and the possibility of designing insurmountable antagonists. Furthermore, because NAMs bind to a different site than orthosteric agonists, they may better block very high-affinity agonists by increasing their disassociation rates even with the orthosteric ligand bound.

### Neutral Allosteric Ligands

NALs bind to an allosteric site without altering receptor activity alone or in the presence of an orthosteric ligand. In principle, NALs could block the activity of endogenous AMs, though this strategy has not yet been widely pursued to date. However, NALs provide useful research tools to verify a lead PAM/NAM mechanism of action by reversing their induced response. Lastly, NALs can provide scaffolds for identifying PAMs or NAMs via structure-activity relationships (SAR) studies that determine how modifications affect bioactivity, bioavailability, and related features.

### Biased-Allosteric Modulators, Ago-Positive Allosteric Modulators, and Probe-Dependent Allosteric Modulators Enable Further Refined Control Over Receptor Activity

Depending on the therapeutic target and disease, one may wish to identify AMs with increasingly specific biological profiles such as allosteric agonists (ago-PAMs) (e.g., Sachpatzidis et al. (2003)), bias AMs (e.g., Trivedi et al. (2009), Goupil et al. (2010)), and probe-dependent AMs (e.g., Chatenet et al. (2013)). Ago-PAMs directly activate the receptor—even in the absence of an orthosteric agonist (Figure 4B, middle right; Table 1). By binding to a less-evolutionarily conserved site, ago-AMs provide a strategy to improve selectivity for targets with closely related subtypes, where binding “off-target” receptors leads to unwanted effects, such as with the muscarinic acetylcholine receptors (mAChRs) (Wootten et al., 2013).

Biased AMs (or biased Ago-PAMs) favor activating one set of pathways—such as the G-protein dependent signaling—but not another—such as β-arrestin dependent signaling, activated by an unbiased orthosteric agonist, typically defined as the endogenous ligand (Figure 1A) (Slosky et al., 2021). Figure 4B (far right) shows a Gα-biased AM that favors Gα-protein signaling over β-arrestin as a representative example; however, β-arrestin-biased AMs are also possible along with, in principle, biased ligands for most other signaling pathways. While this can complicate interpreting *in vivo* results, it also may improve the drug’s biological specificity. Biased AMs are attractive drug targets to precisely hone and modulate endogenous signals (Slosky et al., 2021).

Another way to improve the molecule’s biological specificity is by identifying probe-dependent AMs, which selectively modulate some agonists, but not others (Figure 4B, middle left; Table 1). Probe dependence is a well-established phenomenon for AMs [e.g., Livingston et al. (2018)] and offers the exciting potential to refine a compound’s biological specificity. For example, differentiating between exogenous and endogenous ligands may significantly improve the safety window. Additionally, probe-dependence between two endogenous ligands may favor specific outcomes at a given receptor.

Collectively, peptide/peptidomimetics appear particularly well-suited to achieve these extraordinary biological specificities. The abundance of AM parameters to improve biological specificity makes their pursuit both exciting and challenging in the goal of maximizing drug effectiveness and safety.^3^

## Sources to Identify Protein and Peptide Allosteric Modulator Leads

The potential sources for a lead peptide AM are too numerous to list in their entirety but include both endogenous (Figure 2A) and exogenous sources (Figure 2B). The following section describes established and emerging sources to identify peptide/peptidomimetic AM leads (Figure 2).

### Endogenous Allosteric Modulators Derived From Protein Fragments or “Off-Target” Orthosteric Ligands

Screening biological sources (e.g., tissue or plasma extracts) led to the identification of several endogenous AMs, including P-L-G-NH_2_ **(1)** at the dopamine D2 receptor (Srivastava et al., 1988), 5-HT moduline **(2)** at the serotonin 1B receptor (5-HT_1B_) (Fillion et al., 1996), pepcan12 **(3)** at cannabinoid 2 receptor (CB_2_) (Heimann et al., 2020), and L-V-V-hemorphin-7 **(4)** at the angiotensin II receptor (ATIIR) (Ali et al., 2019) (Figure 2; Table 2). The D_2_ PAM **(1)** was initially isolated from bovine brain tissue during a search for molecules involved in hypothalamic function (Nair et al., 1971). The CB_2_ PAM, Pepcan-12 **(3),** is a fragment of the hemoglobin α-chain isolated from rodent brain, spleen, and adipose tissue using substrate-capture assays (Rioli et al., 2003; Heimann et al., 2020); computational modeling predicts pepcan-12 binds to the extracellular vestibule of the CB2 receptor (Emendato et al., 2018; Heimann et al., 2020). **(2)** is an endogenous NAM of 5-HT_1B_ and was discovered by fractionating rat brain extracts and testing the fractions for affinity at 5-HT receptors (Table 2). Similar techniques identified **(4)** (and related hermorphins) from bovine brain (Karelin et al., 1994). *In vivo* studies showed that **(4)** reduces blood pressure and heart rate in rodent models of hypertension (Cejka et al., 2004), ultimately leading Ali et al. (2019) to show **(4)** is an ATIIR PAM (Ali et al., 2019). These examples collectively show that biological extracts can provide lead peptide AMs relevant to drug discovery/development.

**TABLE 2:** Sequences of linear peptide AMs.

#	Name(s)	Target (activity)	Source	Sequence	References
1	PLG	D_2_ (PAM)	Endogenous	H-PLG-NH_2_	Mishra et al. (1997)
2	5-HT moduline	5-HT2B (NAM)	Endogenous	H-LSAL-OH	Fillion et al. (1996)
3	Pepcan12/RVD-hemopressin	CB_1_(NAM)/CB2 (PAM)	Endogenous	H-RVDPVNFKLLSH-OH	Straiker et al. (2015); Petrucci et al. (2017)
4	LVV-hemorphin-7	ATIIR (PAM)	Endogenous	H-LVVYPWTQRF-OH	Ali et al. (2019)
5	Oxytocin	MOR (PAM)	“Off-target” orthosteric ligand	H-C*YIQNC*PLG-NH_2_	Meguro et al. (2018)
6	Dynorphin A (1-13)	M2R (NAM)	“Off-target” orthosteric ligand	H-YGGFLRRIRPKLK-OH	Hu and el-Fakahany (1993)
7	CXCR4-2-2	CXCR4 (NAM)	CXCR4 TM2	H-LLFVITLPFWAVDAVANWYFGNDD-OH	Tarasova et al. (1999)
8	CCR5-2-1	CCR5 (NAM)	CCR5 TM2	H-LFFLLTVPFWAHYAAAQWDFGDD-OH	Tarasova et al. (1999)
9	CXCR4-2-1	CXCR4 (NAM)	CXCR4 TM2	H-LLFVITLPFWAVDAVANWYFGN-OH	Tarasova et al. (1999)
10	P1pal-12	PAR1 (NAM)	Pepducin	pal-RCLSSSAVANRS-OH	Covic et al. (2002)
11	P1pal-i1-11	PAR1 (NAM)	Pepducin	pal-ILKMKVKKPAV-NH2	Cisowski et al. (2011)
12	p1pal7; PZ-128	PAR1 (NAM)	Pepducin	pal-KKSRALF-OH	Cisowski et al. (2011)
13	P1pal-19	PAR1 (ago-PAM)	Pepducin	pal-RCLSSSAVANRSKKSRALF-OH	Zhang et al. (2015)
14	ATI-2341	CXCR4 (biased)	Pepducin	pal –MGYQKKLRSMTDKYRL-OH	Tchernychev et al. (2010); Quoyer et al. (2013)
15	ATI-2346	CXCR4 (PAM)	Pepducin	pal -KKLRSMTDKYRLH-OH	Tchernychev et al. (2010); Quoyer et al. (2013)
16	ATI-2766, PZ218, x4pal-i1	CXCR4 (NAM)	Pepducin	pal -MGYQKKLRSMTD	Kaneider et al. (2005); O’callaghan et al. (2012)
17	x4pali3, PZ-210	CXCR4 (NAM)	Pepducin	pal -SKLSHSKGHQKRKALK	Kaneider et al. (2005); O’callaghan et al. (2012)
18	P4pal-10	PAR4 (NAM)	Pepducin	pal-SGRRYGHALR-OH	Covic et al. (2002)
19	TAT-h3L4F	5-HT2C (PAM)	Pepducin	H-(YGRKKRRQRRR)PNPDQKPRRKKKEKR-NH2	Ji et al. (2006)
20	r3L4F	5-HT2C (PAM)	Pepducin	Ac-PNPDQKNARRRKKKERR-NH_2_	Anastasio et al. (2013)
21	mF3pal_16	FPR3 (ago-PAM)	Pepducin	pal-KIHKKAFVNSSRPLRV	Lee et al. (2020)
22	ICL3-2b2	ß_2_AR (Gα Biased)	Pepducin	pal-VYSRVFQEAKRQLQKIDKSEGRF-NH_2_	Carr et al. (2014)
23	ICL1-9b2	ß_2_AR (Arrestin Biased)	Pepducin	pal-TAIAKFERLQTVTNYFIT-NH_2_	Grisanti et al. (2018)
24	ICL3-8b2	ß_2_AR (Gα Biased)	Pepducin	pal-LQKIDKSEGRFHV-NH_2_	Carr et al. (2014)
25	ICL1-11b2	ß_2_AR (Arrestin Biased)	Pepducin	pal-TAIAKFERLQTVTNYF-NH2	Carr et al. (2014)
26	ICL1-4b2	ß_2_AR (Arrestin Biased)	Pepducin	pal-VITAIAKFERLQVTN-NH2	Carr et al. (2014)
27	FSHR (271-275)	FSHR (PAM)	Intramolecular Contact	H-YPSHC-OH	Prabhudesai et al. (2021)
28	THG113	FP (NAM)	Intramolecular contact	H-ILGHRDYK-OH	Peri et al. (2002)
29	RSVM	CXCR4 (ago-PAM)	“Bitopic” orthosteric ligand	H-RSVM-OH	Sachpatzidis et al. (2003)
30	ASLW	CXCR4 (ago-PAM)	“Bitopic” orthosteric ligand	H-ASLW-OH	Sachpatzidis et al. (2003)
31	GHRP-6	GhrelinR (PAM)	“Bitopic” orthosteric ligand	H-HwAWfK-NH_2_	Holst et al. (2005)
32	—	Apelin (ago-PAM)	APJR TM2	pal-VTLPLWATYTYR-OH	McKeown et al. (2014)
54	—	β2AR (NAM)	G-protein	H-RDIIQRMHLRQYELL-OH	Boyhus et al. (2018)

Uppercase letters, L amino acids; lowercase letters, D-amino acids; N-terminal groups [pal = palmitate; H‐ = NH_3_^+^. Ac = CH_3_CON]; C-terminal groups [OH = COO^−^; NH_2_ = CONH_2_].

Additionally, endogenous orthosteric agonists at other GPCRs can act as AMs at “off-target” receptors (Figure 2; Table 2). For example, oxytocin **(5)**, an orthosteric agonist for the oxytocin receptor (OTR), is a mu-opioid receptor (MOR) PAM, increasing agonist efficacy in a forskolin-stimulated cAMP inhibition and cellular impedance assay (Meguro et al., 2018). Similarly, Dynorphin A (1-13) **(6)**—the orthosteric agonist at the kappa-opioid receptor (KOR)—is a NAM at the M_2_ receptor that increases the affinity of M_2_ antagonist [^3^H]-methylscopolamine (Hu and el-Fakahany, 1993). It is unclear if these two examples are the exception or the rule; future work looking at known endogenous ligands at “off-target” receptors may reveal more endogenous AMs and drug discovery leads.

### Protein-Protein Interactions between the Receptor and a Transducer or Accessory Protein as Allosteric Modulator Leads

Many protein-protein interactions act as endogenous protein AMs and offer opportunities to identify peptide AM sequences that bind to an allosteric site (Figure 1B). Notably, G-proteins are quintessential PAMs binding to the intracellular receptor face to stabilize a “high-affinity” R_a_ conformation and increase orthosteric agonist affinity via “positive cooperativity.” Receptor contacts with transducer proteins (e.g., Gα (Dutka et al., 2019; Syrovatkina and Huang, 2019), β-arrestin (Shukla et al., 2013))—and accessory proteins (e.g., receptor complement proteins (RCPs) (Routledge et al., 2020), receptor activity modulating proteins (RAMPs) (J Gingell et al., 2016), GPCR oligomers (Borroto-Escuela et al., 2013; Pin et al., 2019), receptor tyrosine kinases (RTKs) (Di Liberto et al., 2019), the protein phosphatase and tensin homolog (PTEN) (Anastasio et al., 2013), and others (Pasternak, 2017))—allosterically regulate receptor function. Therefore, these endogenous protein AMs can provide valuable lead peptide AM sequences, often with available structural, biochemical, and computational information to guide lead development/optimization (Figure 2).

While many of these targets remain untapped, several proof-of-concept examples exist showing that protein-protein interactions can provide AMs, including the α-helix of Gα (Boyhus et al., 2018) and oligomers of GPCRS [e.g., Kabli et al. (2014)]. Importantly, evolution already honed many of these endogenous AMs, providing rich structural and SAR information for drug discovery and development. For example, the α-helix 5 of Gα, which contacts the receptor, contains a set of highly conserved residues likely important for receptor binding and a second set of variable residues that likely govern receptor selectivity (Flock et al., 2015), providing a great starting point for SAR studies. Likewise, disruption of oligomer contacts may explain the NAM activity of CXCR4-2-2 **(7)** CCR5-2-1 **(8)** and CXCR4-2-1 **(9)**, derived from TM2 of CXCR4 and C-C chemokine receptor 5 (CCR5). Both sequences block orthosteric agonist-mediated signaling and HIV entry in a cell model assay (Tarasova et al., 1999) (Table 2). In another AM protein-protein interaction example, sequences derived from the predictive interface of the MOR-delta opioid receptor (DOR) heterodimer (MDOR) may act as AMs, with sequences from TM1 of MOR modulating orthosteric DOR agonist activity (He et al., 2011) and the sequence of intracellular loop 4 (ICL4) of DOR perhaps modulating MOR orthosteric agonist activity (Kabli et al., 2014). Disentangling heteromeric- and allosteric-mediated effects is often tricky as bivalent orthosteric ligands can produce similar effects [e.g., Olson et al. (2018); Keresztes et al. (2021)]; however, the design principles remain the same and are often a distinction without a difference, in so far as developing drugs with a desired biological effect.

The protein-protein interactions that allosterically modulate GPCRs are extensive and diverse, as noted above. The rapidly growing number of GPCR crystal structures and other molecular studies revealing key contacts between receptors and AM proteins provide excellent leads with highly relevant structural data typically unavailable for small molecules early on in discovery that in principle can reduce time and cost of future development. Another valuable resource for identifying protein-protein interactions is the freely accessible protein common interface database (ProtCID) (Xu and Dunbrack, 2020) that provides a starting point for medicinal chemists during the early phases of drug discovery.

### Intramolecular Contacts Are Derived From the Intracellular (i.e., Pepducins), and Extracellular Loops as Allosteric Modulator Leads

In addition to intermolecular protein-protein interactions, intramolecular contacts can provide AM leads too. The most prominent example is the pepducins, a family of AMs derived from the intracellular loops/tail (ICL1-ICL4) of GPCRs attached to lipid or steroid, such as palmitate, myristate, or lithocholic acid (Figure 2A) (Carlson et al., 2012). Pepducins typically bind to other ICLs at the intracellular face to stabilize a specific conformation [e.g., Covic et al. (2002), Kuliopulos and Covic (2003), and Janz et al. (2011)], though other mechanisms may exist, such as inhibiting protein-protein interactions. The attached lipid moiety facilitates membrane insertion followed by “flipping” the peptide across the bilayer to the cytosolic side, improving membrane permeability and peripheral bioavailability [e.g., Covic et al. (2002), Kuliopulos and Covic (2003), Tsuji et al. (2013), and Zhang et al. (2015)] (Figure 3C). Reported pepducin AMs include P1pal-12 **(10)**, P1pal-i1-11 **(11),** and p1pal7 (or PZ128) **(12)** at the PAR1 (; Kuliopulos and Covic, 2003; Leger et al., 2006; Cisowski et al., 2011; Zhang et al., 2012; Gurbel et al., 2016; Kuliopulos et al., 2020); P1pal19 **(13)**, ATI-2341 **(14)**, ATI-2346 **(15)**, PZ218 **(16)**, and PZ210 **(17)** at CXCR4 (Tchernychev et al., 2010; Janz et al., 2011; O’Callaghan et al., 2012; Quoyer et al., 2013); P4pal-10 **(18)** at the protease-active receptor 4 (PAR4) (Leger et al., 2006; Carr et al., 2016; Holdfeldt et al., 2020); TAT-h3L4F **(19)** and r3L4F **(20)** at the 5HT_2C_R (Anastasio et al., 2013; Ji et al., 2006); mF3pal_16 **(21)** at formyl peptide receptor 3 (FPR3) (Lee et al., 2020); and ICL3-2 **(22)**, ICL1-9 **(23)**, ICL3-8 **(24)**, ICL1-11 **(25)**, and ICL1-4 **(26)** at the β2-adrenergic receptor (ß_2_AR) (Table 2) (Carr et al., 2014; Grisanti et al., 2018) and even led to **12** entering clinical trials (Kuliopulos et al., 2020).

Similarly, extracellular loops (ECLs) produce intramolecular contacts with other residues and can provide AM leads, including at the human follicle-stimulating hormone receptor (hFSHR) (Prabhudesai et al., 2021), prostaglandin F2α (PGF2α) receptor (FP) (Peri et al., 2002), E_2_ prostaglandin receptor 4 (EP4) (Leduc et al., 2013), and the vasopressin 2 receptor (V_2_R) (Rihakova et al., 2009). For example, computational modeling predicted a 5-amino acid ECL sequence of human follicle-stimulating hormone receptor (hFSHR) 271-275 **(27)** could bind the ECLs and stabilize the FSHR active conformation. Follow-up studies confirmed **(27)** as a PAM *in vitro*—promoting binding of the endogenous agonist, FSH and cAMP production—and *in vivo*—increasing granulosa cell proliferation and ovarian weight gain mediated by FSH (Table 2) (Prabhudesai et al., 2021). In another example, THG113 **(28)** is a NAM designed by Theratechnologies derived from ECL2 of the prostaglandin F2α (PGF2α) receptor (FP), which inhibits preterm labor in rodent models (Peri et al., 2002) (Table 2). Such intramolecular contacts that replicate known (or predicted) interactions to stabilize a receptor conformation enable lead identification with the design of small target-based libraries [e.g., Edwards et al. (2007), Boyhus et al. (2018)], with significantly higher hit rates than small-molecule HTS libraries.

### Allosteric Modulator Leads From Endogenous “Bitopic” Ligands

Many endogenous orthosteric peptide agonists are effectively bitopic ligands, forming primary contacts with the orthosteric sites and secondary contacts to the ECL allosteric sites (Figure 2). For example, at the C-X-C chemokine 4 receptor (CXCR4), the endogenous agonist C-X-C chemokine ligand 12 (CXCL12) contacts the classical orthosteric TM site and the allosteric ECLs (Adlere et al., 2019). As such, Sachpatizidis et al. (2003) used CXCL12 as a lead to identify the ago-PAMs R-S-V-M-OH **(29)** and A-S-L-W-OH **(30)** from a library of mutations to the 4 N-terminal amino acids. **(29)** and **(30)** putatively bound to the ECLs and did not compete with an orthosteric antagonist (Sachpatzidis et al., 2003). Another example, at the urotensin II receptor (UTS2R), converted the endogenous agonist, urotensin related peptide (URP), into a NAM after pharmacophore minimization and scaffold substitution (Chatenet et al., 2013; Douchez et al., 2017; Dufour-Gallant et al., 2015). Similarly, a truncation of the endogenous ghrelin agonist, growth hormone-related peptide 1-28 (GHRP (1-28)) led to the AM GHRP6 **(31)**, which decreases GHRP (1-28) potency ∼5-fold but increases its efficacy by ∼150% in IP accumulation assays at growth hormone secretagogue receptor _1A_ (GHS_1A_R or Ghrelin receptor) (Table 2) (Holst et al., 2005). Mutagenesis studies and computational models suggest GHRP-6 (and other small molecule Ghrelin receptor AMs) occupies similar ECL sites, which partially—but not completely—overlap with the GHRP1-28 site (Holst et al., 2009). Most peptide receptors have many endogenous biologically active fragments of varying length and may provide useful starting points for AMs, especially when one can easily separate the “orthosteric” and “allosteric” pharmacophores.

Ironically, many of these discoveries appear serendipitous, suggesting AM screens of endogenous protein fragments, endogenous ligand libraries at “off-target” receptors, or endogenous orthosteric “bitopic” ligands may prove particularly useful for lead identification.

### Antibodies, Autoantibodies, and Nanobodies as Potential Peptide Allosteric Modulator Leads

Many antibodies act as AMs, recognizing their targets (epitopes) with relatively short and diverse amino acid sequences called complementary determining regions (CDRs), with the remainder of the protein providing immunoregulatory functions (Figure 2). Antibodies, due to their membrane impermeability and preference to bind linear epitopes, typically recognize ECLs. Notably, chronic diseases produce autoantibodies against 20+ GPCRs, which usually bind to the ECLs acting as PAMs or ago-PAMs (Skiba and Kruse, 2021). These known sequences could provide leads for NALs that block an autoantibody’s ago-PAM or PAM activity contributing to the disease-state. Likewise, engineered antibodies can provide leads from diverse sequence libraries (Hutchings, 2020).

Nanobodies—truncated single domain antibodies derived from camels—also act as AMs, with PAMs shifting orthosteric agonist affinity up to 15,000-fold [e.g., Staus et al. (2016), Heukers et al. (2019)]. Nanobodies are particularly useful to distinguish between the R_I_ and R_A_—as AMs must do—because their CDRs form a convex β−loop that can interact with conformationally dependent 3D geometries (Manglik et al., 2017). Additionally, nanobodies are easily expressed heterologously, facilitating screening against intracellular targets. A good starting place for sequence and structural information of nanobodies is the free database, “Institute Collection and Analysis of Nanobodies (iCAN) [http://ican.ils.seu.edu.cn] (Zuo et al., 2017).

Since antibodies and nanobodies inherently evolve to bind specific epitopes, they fittingly enable using directed-evolution platforms to refine lead sequences, such as the Viral Evolution of Genetically Actuating Sequences (VEGAS) platform for intracellular nanobodies (English et al., 2019) and related techniques (Maeda et al., 2018). These directed-evolution platforms are exciting as a way forward in AM drug development since, unlike small molecule libraries, directed evolution can provide “intelligent” combinatorial libraries early in development with millions of possible combinations.

Converting CDRs to peptidomimetics can decrease their size, lower production cost, improve/alter bioactivity, enhance drug-like properties (e.g., membrane permeability), and reduce potential immunogenic concerns (Murali and Greene, 2012). While no one has yet developed a GPCR AM peptidomimetic from a CDR to our knowledge, the strategy has worked at numerous other antibody targets (Murali and Greene, 2012). Whether further development (e.g., nanobody → peptidomimetic) is warranted depends on the target, disease, bioactivity, route of administration, and related considerations, as biologics can provide therapeutics without further modifications. However, nanobodies binding to intracellular targets typically cannot cross the membrane and thus provide a particularly intriguing platform for peptidomimetic development. Nonetheless, antibody and nanobody CDR sequences provide an excellent untapped source for AM leads.

### Exogenous Synthetic or Virtual Allosteric Libraries to Identify Lead Allosteric Modulators

Several virtual databases or exogenous libraries provide useful information for peptide AM lead identification. The freely available “allosteric database” (ASD, available at http://mdl.shsmu.edu.cn/ASD), provides powerful tools, such as the “AlloSite,” “Allosite-Potential,” AlloFinder,” “Allo-Pathway,” and “Allosterome” with avenues for lead identification from predicted or established allosteric interactions, including protein-protein interactions and small peptides (Liu et al., 2020). For example, an ASD search of GPCR AMs classified as “polypeptides” revealed 420 hits. When a lead’s secondary structure is known or predicted, synthetic libraries can help identify peptidomimetic leads using ligand-based drug discovery and classical screening approaches [e.g., Whitby et al. (2011)]. Furthermore, larger exogenous synthetic libraries also provide excellent sources, similar to small-molecule HTS libraries, especially if no lead is available from the aforementioned sources. For example, a DNA-encoded library (DEL) screened against the purified ß_2_AR identified small-molecule-like peptidomimetics with PAM and NAM activity (Figure 2) (Ahn et al., 2017; Ahn et al., 2018). The fact that both hits contained 2 or more amide bonds (e.g., they are peptides) makes it tempting to speculate that peptide motifs can access chemical space particularly well suited for AM development.

Exogenous libraries can come from nature-derived sources—such as the conopeptides derived from marine snail, scorpion, snake venom, or cyclotides from plants—which all consist of stable structural motifs considered “privileged scaffolds” with access to different chemical space than synthetic libraries (Sharpe et al., 2003; W. Gruber et al., 2010; Ragnarsson et al., 2013; Muratspahić et al., 2019). Such sources have produced several AMs, including Vc1.1 at the GABA_B_ (Clark et al., 2006), ρTIA at the α_1B_ adrenergic receptor (α_1B_AR) (Sharpe et al., 2003), and muscarinic toxin 7 (MT7) at the muscarinic acetylcholine receptor 1 (M_1_AChR) (Maeda et al., 2020). Further, genetically encoded cyclic peptide libraries are available in various formats, including phage display, mRNA display, and split-intein circular ligation of peptides and proteins (SICLOPPS) (Valentine and Tavassoli, 2018; Sohrabi et al., 2020). However, most of these technologies require purified protein or bacterial expression of the target, complicating their application for GPCR drug discovery. On the other hand, exogenous synthetic libraries with 10^8^ combinations are possible and exploit nonnatural amino acids providing access to more chemical space than genetically encoded libraries (Quartararo et al., 2020). While these genetically encoded libraries have not been applied to AMs yet, they offer established sources for drug leads in general.

## The Advantages and Disadvantages of Peptide/Peptidomimetic AMs

### Peptide’s Advantages and Disadvantages Arise From Their Inherent Chemical Properties

Peptidomimetics is a broad field of medicinal chemistry that has led to several clinically approved drugs (Qvit et al., 2017). Inherently, the advantages of peptides—such as their high affinity and high selectivity—arise from their chemical properties, as do their disadvantages—such as metabolic instability and low bioavailability. Peptides contain a polyamide “backbone” resulting in a repeating -NH-Cα-CO- motif (Figure 5A, cyan). Intramolecular H-bonds between the backbone N-H donors and C=O acceptors stabilize the secondary structures—α-helices, β-turns, γ-turns, and so on—and provide a scaffold to project Cα R-groups or sidechains (Figure 5A, yellow). The orientation of the flexible backbone dihedral ϕ and ψ angles (e.g., Ramachandran plots) governs a sequence’s possible secondary structures, while the dihedral χ angle(s) determine the sidechain orientations in space. The χ angle(s) facilitate a peptide’s high-affinity and selective interactions at a target site, making their restraint a useful consideration during lead optimization of bioactivity (Hruby et al., 1997). The modularity of peptides also enables well-established synthetic schemes, including solid-phase peptide synthesis (SPSS), which is easy and automatable, with thousands of commercially available building blocks [e.g., Mäde et al. (2014)].

However, the flexibility, modularity, and H-bond network that stabilize secondary structures of biological significance comes with disadvantages. Peptide’s inherent flexibility, numerous H-bond donors/acceptors, and multiple charges contribute to their low bioavailability and inability to cross membranes making oral bioavailability or engaging intracellular targets difficult without further modifications. Moreover, their metabolic instability primarily arises from 4 motifs on the peptide: 1) and 2) NH_3_^+^ and COO^−^ termini, 3) amide bonds, and 4) sidechain metabolites (Figure 5A, yellow). Peptidomimetics address these disadvantages and retain peptide’s advantages by identifying the minimum pharmacophore, stabilizing the bioactive conformation via restricting χ, ϕ, and ψ angles, and performing SAR studies to improve bioactivity, bioavailability, and membrane permeability (Figure 3).

### Peptidomimetics Combine the Advantages of Small Molecules and Peptides

Here, we define peptidomimetics as molecules that combine peptides’ and small molecules’ desirable traits by mimicking a peptide’s bioactive pharmacophore while improving its drug-like properties, with a generic example of the key steps shown in Figure 3. For our purposes, “small-molecule peptidomimetics” focus on converting peptides to small molecules that approximately meet Lipinski’s rule of 5 (<500 kDa; logP <5; <10 H-bond acceptors; <5 H-bond donors), whereas “peptide-like peptidomimetics” retain higher molecular weight and more endogenous amino acids. To describe peptidomimetic modifications, we use a slightly modified nomenclature introduced by Lenci and Trabocchi (2020) based on the location of the synthetic alteration(s) (Lenci and Trabocchi, 2020). “Local modifications” are changes to 1 (or 2 sequential) amino acids, such as replacing amides and sidechains with bioisosteres (Figure 6); dipeptide secondary structure mimetics, such as those to stabilize β-turns; or conjugating moieties to promote specific properties (e.g., increase solubility and/or membrane permeability) (Figure 5B). On the other hand, “global modifications” connect two nonsequential residues, which typically improve compound metabolic stability and stabilize bioactive secondary structure conformations—such as an α-helix or β−loops (Figures 5C,D). While certain modifications tend to alter certain characteristics (e.g., bioavailability or bioactivity), in principle, most modifications can alter either.

Further, peptide/peptidomimetic leads can provide several advantages over small-molecule hits. First, since many protein-protein interactions are known AMs, crystal structures and other structural information are often available to guide development. Second, peptides often provide better bioactivity earlier in the process from a smaller number of screened molecules since their structure was refined during evolution (e.g., Gα contacts with a receptor); relatedly, such examples provide putative allosteric sites, which is useful during lead optimization and target verification. Fourth, identifying a peptide pharmacophore is (usually) straightforward due to their modularity and well-studied structural motifs (Figures 3A,B). Allosteric modulators typically have steep SARs; therefore, many small-molecule AM projects employ combinatorial approaches that produce leads which an iterative process would have missed [e.g., Lindsley et al. (2016)]; notably, peptides’ modularity makes them particularly well-attuned for combinatorial chemistry [see Lam et al. (1991), Mäde et al. (2014), Bozovičar and Bratkovič (2019)].

## Overview of Peptide and Peptidomimetic Modifications to Improve AM Bioactivity and Drug-Like Properties

### Minimizing the Peptide Lead Sequence and Bioactive Pharmacophore

Typically, the first step in peptidomimetic design is to minimize and identify the peptide sequence’s pharmacophore through systematic truncations, deletions, and amino acid scans (Figure 3A) (Hruby et al., 1997; Jamieson et al., 2013). Amino acid truncations remove each N- and C-terminal amino acid one by one, followed by similar deletion scans that systematically remove each remaining amino acid to identify the necessary sequence for bioactivity. Classically, the next step in pharmacophore identification is determining the required stereocenters and side chains for bioactivity (Figure 3). Since endogenous amino acids, except glycine (G), are generally L* stereoisomers, a D*-amino scan can determine the critical stereocenters for the bioactive conformation. Similarly, an alanine-scan—introducing the simple CH_3_ side chain at each position—can reveal the necessary functional groups for bioactivity (Figure 3A).

Other scans can elucidate additional pharmacophore information, including N-alkyl amino acid scans to determine the importance of amide protons for H-bonding or promoting extended conformations (Sagan et al., 2004). On the other hand, when investigating β-turns, proline (P), and lactam-scans can determine the optimal ϕ and ψ angles for bioactivity (Figure 5A) (Jamieson et al., 2013). For α-helical structures, stapled and bridged peptide scans can determine the optimal positions to place the side chain bioisosteres to form a covalent bond that stabilizes the helical macrocycle (Jamieson et al., 2013). When possible, biophysical experiments (e.g., nuclear magnetic resonance (NMR) or x-ray crystallography) or computational methods (e.g., conformational or docking) should inform design. While one can use iterative biophysical experiments at any stage, structural investigations are instrumental when designing small molecule peptidomimetics, as shown in Figure 3D.

#### ß_2_AR, APJR, and PAR1/4 Allosteric Modulators: The Length and Source of Peptide AM Leads Can Alter Activity, Signaling Bias, and Subtype Selectivity

One advantage of leads from known sources—such as pepducins or protein-protein interactions—is the ability to perform “sequence scans” that often yield multiple AMs with diverse and specific biological activities. Sequence scans combine lead identification and pharmacophore minimization steps to generate a small peptide library of various lengths and sequences. For example, McKeown et al. (2014) generated a 369-compound library from apelin receptor (APJR) sequence using consecutive and overlapping 12 residue peptides. This approach identified a TM2 sequence pal-V-T-L-P-L-W-A-T-Y-T-Y-R-OH **(32)** as a potent ago-PAM, which a more targeted scan would have missed (Table 2). Using a similar strategy, Carr et al., (2014) generated 51 ß_2_AR pepducins (∼14-20 residues long) based on sequences of ICL1, ICL2, and ICL3 (Carr et al., 2014). ICL3-2 **(22)** and ICL3-8 **(24)** were derived from ICL3 and acted as Gα_s_ biased ago-PAMs with potential as asthma treatments (Table 2) (Carr et al., 2014). **(24)** is a truncated version of **(22)** with similar activity, akin to truncation studies (Figure 3A). On the other hand, several ICL1 pepducins, such as ICL1-9 **(23)**, ICL1-11 **(25)**, and ICL1-4 **(26)** produced β-arrestin2 biased ago-PAMs. Further work at other receptors is required to see if ICL1 and ICL3 produce Gα and β-arrestin biased ago-PAMs consistently or if it is receptor-dependent. These examples show “sequence scanning” facilitates the identification of AM leads with diverse biological profiles at different receptor sites from small directed “libraries” with substantially higher hit rates than small molecule HTS libraries.

Perhaps, not surprisingly, several of these pepducins activated Gα_s_ in a receptor-independent manner—a phenomenon seen in other pepducin studies (Carr et al., 2016)—indicating the importance of counter screening early in lead identification. However, identifying AM targets is not a peptide-specific problem, as these difficulties can plague small molecule HTS hits as well. In fact, peptide leads often have a putative site (e.g., pepducins at the intracellular face), making site verification significantly easier.

During the early phases of lead development and pharmacophore identification, it is critical to screen for subtype selectivity and different AM profiles, if possible, to avoid missing important SAR features. For example, NAMs derived from their respective ICL3 loops of PAR1 and PAR4—P1pal-12 **(10)** (Covic et al., 2002) and P4pal-10 **(18)** (Covic et al., 2002), respectively—show different PAR1/PAR4 selectivity ratios. **(10)** selectively blocks PAR1 signaling, but **18** blocks both PAR1 and PAR4 signaling as measured by intracellular Ca^2+^ and platelet aggregation assays. **(13)**—with 7 more C-terminal amino acids than **(10)**—is an ago-PAM, again indicating the sequence length can convert the AM activity (Table 2). Lastly, mutating several arginine (R) or lysine (K) residues to serine (S) on P1pal-19 **(13)** significantly reduced ago-PAM activity, showing the necessity of the positive charges. Collectively, these studies at PAR1, ß_2_AR, and APJR using sequences derived from putative intramolecular contacts demonstrate these sources’ ability to produce AMs with diverse pharmacological profiles.

#### 5-HT_1B_ and FP Allosteric Modulators: D-amino Acid and Alanine Scans Reveal Pharmacophores of 5-HT Moduline and THG113

**FIGURE 7:**
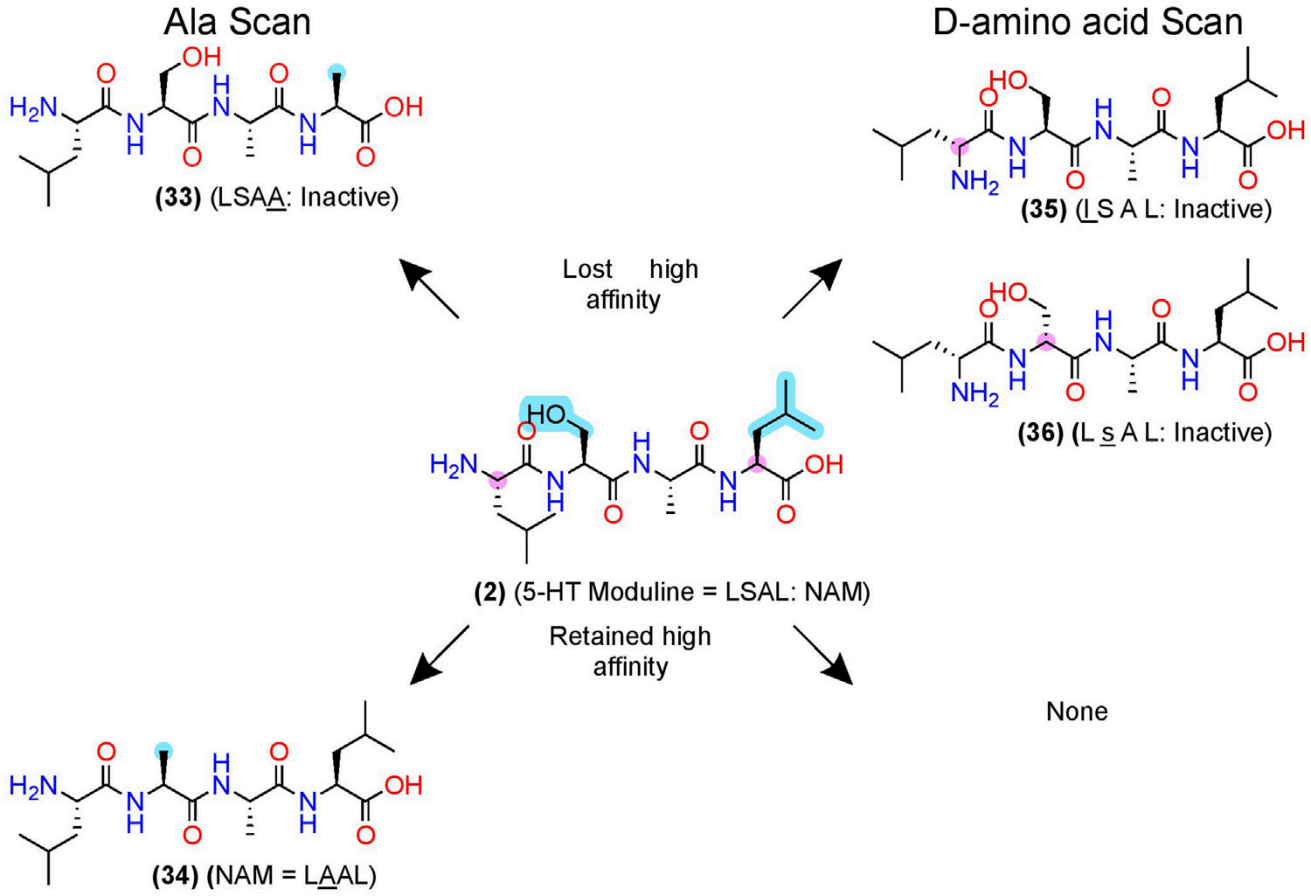
D-amino acid and alanine scans for a 5HT_1B_ NAM—5-HT-moduline. **(Left)** the key pharmacophore features are highlighted in yellow as determined via an alanine scan. **(Right)** D-amino acid scan. (Top) examples of modifications that lost NAM function or (bottom) retained NAM activity. Structures from Plantefol et al. (1999). Uppercase letters indicate L-amino acids, and lowercase letters represent D-amino acids. The in-text compound number is bold (original name: activity). Sidechain pharmacophore features are labeled in cyan and backbone features labeled in pink. Figure was created with Chemdraw20.0.

After pharmacophore minimization, an alanine- and D-amino acid scan can further refine the lead’s key structural and chemical pharmacophore features [e.g., Plantefol et al., 1999] (Figure 3). In one example, Fillion et al. performed an alanine and D-amino acid scan for 5-HT moduline **(2)**, an endogenous NAM tetrapeptide–L-S-A-L-OH–at the 5-HT_1B_R (Fillion et al., 1996), which reduces binding of the orthosteric agonist serotonin (Massot et al., 1996) (Figure 7). The alanine-scan demonstrated that position 4 required a larger hydrophobic amino acid as a leucine (L) → alanine (A) substitution, **(33)**, eliminated the high-affinity binding (Figure 7, upper left). At position 2, substituting the S→A in **(34)** did not significantly alter [^3^H]5HT-Moduline binding (Figure 7, lower left), indicating future studies could modify the S to improve drug-like properties. Furthermore, S→P substitution did not alter affinity, indicating the backbone H-bond donors did not contribute to affinity either. Lastly, the D-amino scan revealed chirality is important at all 4 positions, with l (D-Leucine)**—(35)—**and s (D-Serine)—**(36)**—at the 1 and 2 positions, nearly abolishing affinity. When D-amino acid substitutions retain activity, those analogs can also increase metabolic stability by reducing protease recognition. The 5-HT-moduline **(2)** studies show a great concise example of the information providing from alanine and D-amino acid scans.

**FIGURE 8:**
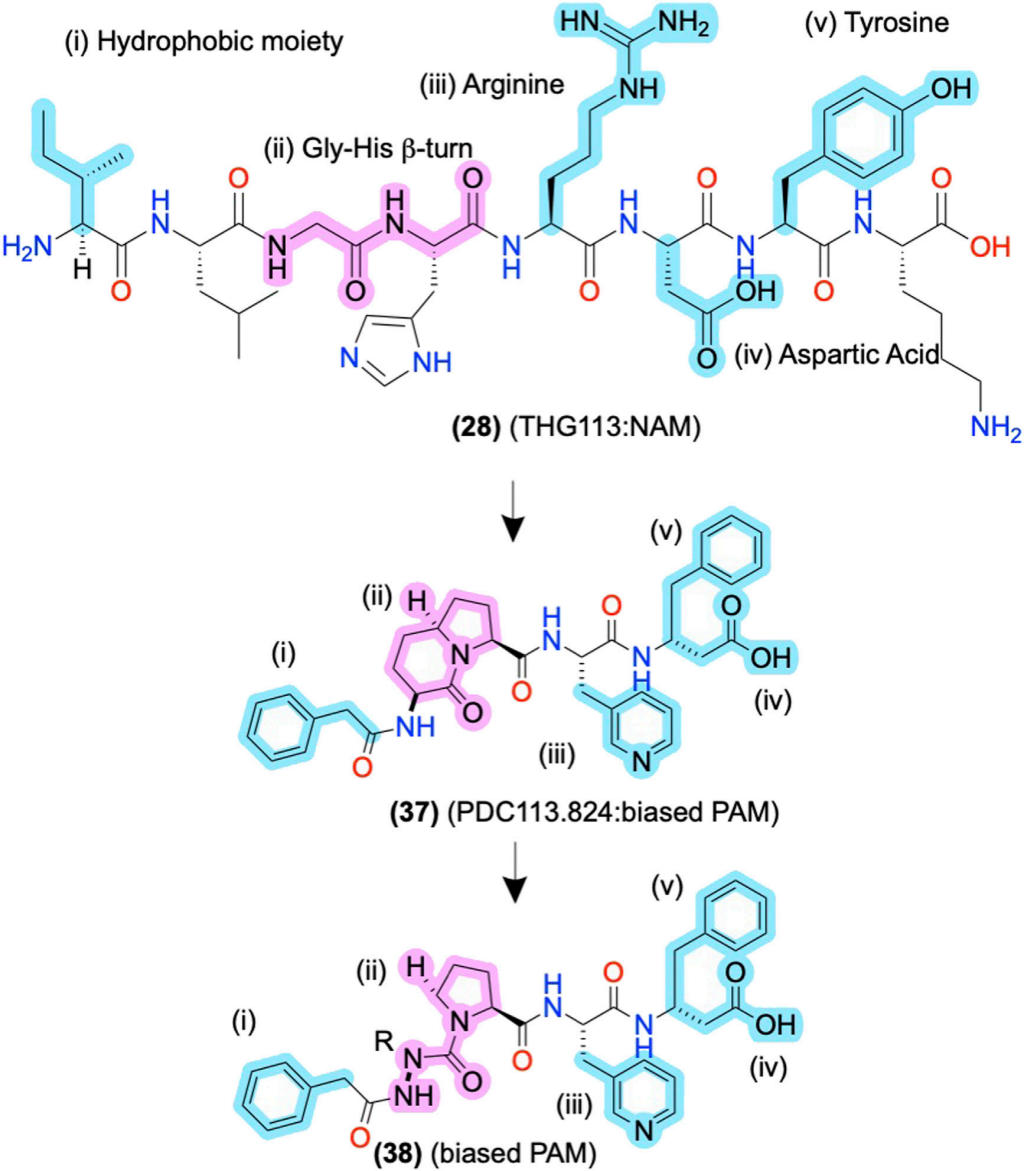
ECL-derived sequences and peptidomimetic design of NAMs and biased-PAMs at FP. The key pharmacophore features are highlighted in yellow and numbered i, ii, iii, and iv, showing the peptidomimetic development to improve bioactivity, conformational stability, and metabolic stability. The in-text compound number is bold, followed by original name: activity. Structures are originally reported by Peri et al., 2002; Bourguet et al., 2009; and Goupil et al., 2010. Sidechain pharmacophore features are labeled in cyan and backbone features labeled in pink. Figure was created with Chemdraw20.0.

The initial alanine and enantiomeric scans inform modifications beyond their initial substitutions shown in **(2)**, above (Figure 3), as the peptidomimetic development of THG113 **(28)** shows. **(28)** is a NAM derived from ECL2 of the FP, which inhibits preterm labor in rodent models (Peri et al., 2002) (Figure 8). Intriguingly, Lubell and colleagues report several ECL-derived AMs that substitute all the endogenous L-amino acids with D-amino acids (Peri et al., 2002; Rihakova et al., 2009; Leduc et al., 2013). However, it is unclear why the enantiomeric sequences routinely work or whether the L-amino acid sequences produce comparable results. Nonetheless, Lubell and colleagues performed enantiomeric and alanine scans of **(28)** to identify its key pharmacophore substituents as i) an N-terminal hydrophobic moiety (cyan), ii) a β-turn around the glycine-histidine (-G-H-) motif (pink), and ii/iii/iv) the arginine-aspartic acid-tyrosine (-R-D-Y-) side chains (cyan) (Figure 8) [as described in Peri et al. (2002), Bourguet et al. (2009), Goupil et al. (2010)]. Using this pharmacophore, further modifications led to PDC113.824 with putative improved metabolic stability and bioavailability **(37)**, in which i) a benzyl replaced the N-terminal hydrophobic moiety, (ii) indolizidine mimic replaced the G-H β-turn, ii) a pyridylalanine replaced the R, and (iv/v), the D-Y motif turned into C-terminal β-phenylalanine (Figure 8) (Goupil et al., 2010). **(37)** is a biased PAM that potentiates PKC and ERK1/2 signaling but reduces Gα_12_ signaling of the endogenous orthosteric agonist PGF2α, leading to the selective inhibition of myometrial contractility via NAM activity for Gα_12_ (Goupil et al., 2010). Therefore, **(37)** provides another example of peptidomimetic AMs producing incredible biological specificity.

### Amide- and Sidechain-Bioisosteres to Improve Bioactivity, Metabolic Stability, and Bioavailability

After identifying the pharmacophore, SARs with amide- and sidechain-bioisosteres can improve metabolic stability while retaining (or improving) the electronic and physiochemical properties necessary for bioactivity (Figures 3B, 6). Local modifications can also stabilize bioactive conformations [e.g., Lenci and Trabocchi (2020)] through restraining ϕ and ψ angles, often informed by computational and biophysical studies. Depending on the peptide’s size, physicochemical properties, and end goal, the structural information gained from the SARs can inform small molecule identification with ligand-based computational approaches, such as through pharmacophore search, scaffold replacement, or docking studies (Figure 3D).

#### D_2_ and FP Allosteric Modulators: Amide Isosteres to Improve Activity and Stabilize the Bioactive Conformation

**FIGURE 9:**
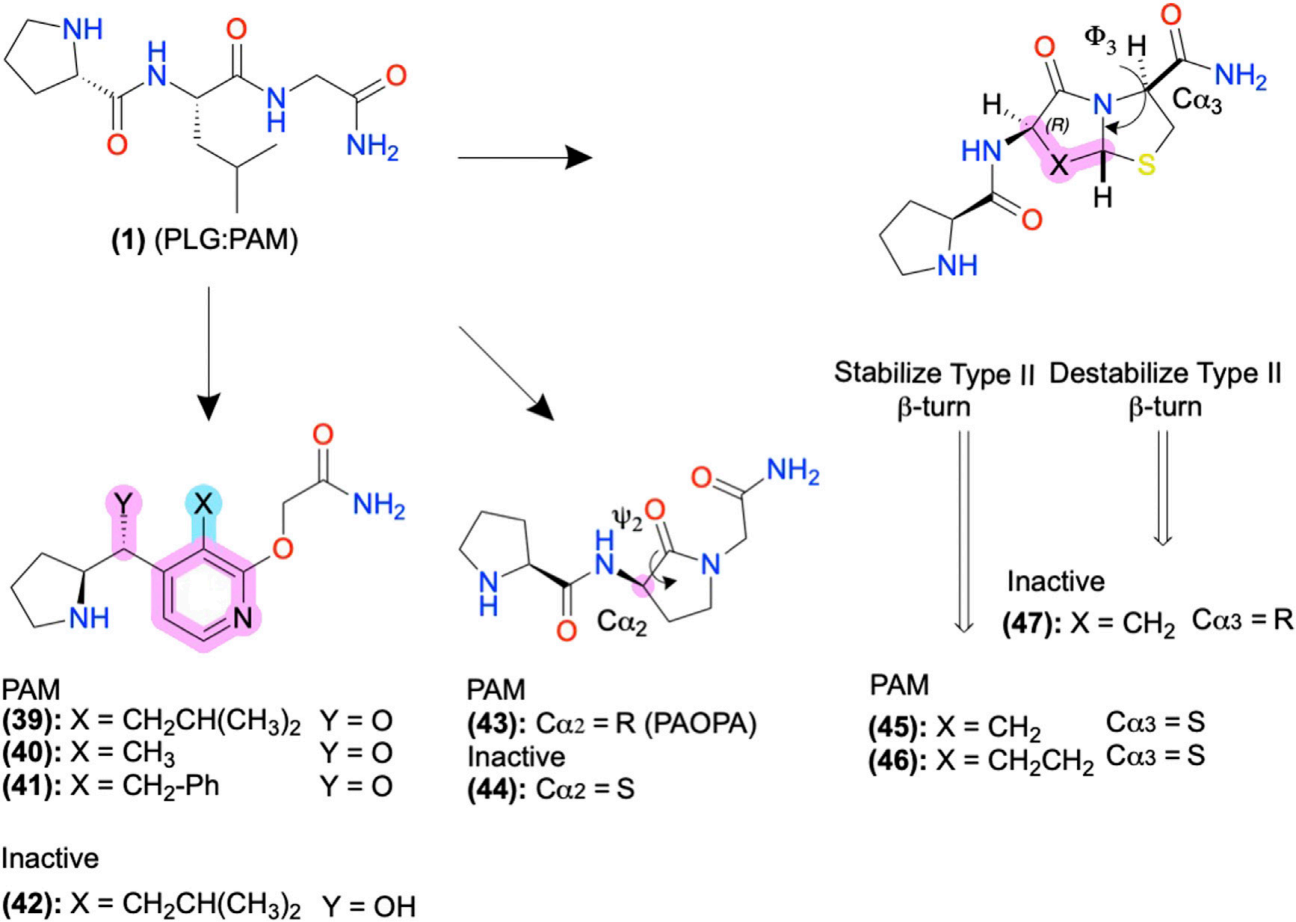
D_2_ AMs designed from H-PLG-NH_2_ to constrain a type II β-turn. Restricting the ψ_2_ and ϕ−_3_ angles stabilizing the type II β-turn improved PAM activity, and destabilization decreased PAM activity. Backbone modifications were highlighted in pink, and side-chain modifications were highlighted in cyan. The in-text compound number is bold, followed by original name: activity. Structures were originally reported by Yu et al., 1988; Sreenivasan et al., 1993; Subasinghe et al., 1993; Saitton et al., 2004; Saitton et al., 2008; and Bhagwanth et al., 2013. Figure was created with Chemdraw20.0.

In FP AMs derived from **(37)**, the bicyclic moiety was replaced with an aza-glycine to produce **(38),** which retained its NAM activity and extended delivery time in a murine preterm-birth model (Figure 8) (Bourguet et al., 2011; Mir et al., 2019). Several SAR series of the endogenous D_2_ PAM tripeptide P-L-G-NH_2_ **(1)** provides examples of amide bioisosteres to improve bioactivity and metabolic stability (Figure 9). For instance, replacing the 2nd position -L- with a pyridine analog **(39)**, constraining the Cα-NH bond, improved PAM activity compared to **(1)**, as measured by increased efficacy of the orthosteric agonist, N-propylapomorphine (NPA) (Figure 9) (Saitton et al., 2004). On the other hand, **(40)** and **(41)** showed activity similar to **(1)**, indicating the importance of the -L- side chain (X). **(42)** substituted carbonyl (y = O) with a hydroxyl (y = OH) and did not show significant PAM activity suggesting the pharmacophore may require the H-bond acceptor (Saitton et al., 2008). Beyond these examples, a wide range of backbone and amide bioisosteres exist to restrain the ligand’s conformation, reduce metabolic instability, and refine sidechain orientations to precisely refine peptidomimetic bioactivity and stability, with examples for phenylalanine (F) shown in Figures 6B,C and reviewed extensively by Lenci and Trabocchi (2020).

#### D_2_ Positive Allosteric Modulators: Biophysical Studies to Guide Structural Restraints and Improve Bioactivity

The D_2_ PAM P-L-G-NH_2_ **(1)** primarily adopts a type II β-turn conformation according to computational, NMR, and crystallography studies (Reed and Johnson, 1973; Bhagwanth et al., 2013). Therefore, Johnson and colleagues designed analogs of **(1)** to stabilize or destabilize the secondary structure through the restraint of the -L- ψ_2_/ϕ_2,_ and -G-NH_2_ ψ_3/_ϕ_3_ to determine the bioactive conformation (Figures 5A, 9). Introducing a lactam to constrain ψ_2_ produced the R isomer PAOPA **(43)** with a ∼1,000-fold increase in PAM potency over **(1)**, as determined by shifts in radiolabeled agonist binding (Yu et al., 1988). **(44)** converted the Cα_2_ chiral center R→S to destabilize the turn, which eliminated the AM activity, suggesting the type II β-turn is the bioactive active conformation (Figure 9, pink) (Yu et al., 1988; Sreenivasan et al., 1993; Bhagwanth et al., 2013). Next, the introduction of a bicyclic motif to constrain ϕ_3_ led to the active PAM’s **(45)** and **(46)**, which adopt the type II β-turn in NMR studies (Figure 9) (Subasinghe et al., 1993). In another stereoisomer study at Cα_3_, converting the S isomer → R isomer produced the inactive **(47)**, again supporting the β-turn hypothesis as turn destabilization coincided with losing activity. While in many cases, the β-turn appears necessary for bioactivity, exceptions exist indicating multiple secondary conformations can produce acceptable 3D pharmacophores or multiple bioactive conformations exist (Raghavan et al., 2009).

*In vivo* testing showed **(43)** potentiates orthosteric D_2_ agonist-mediated behavior with ∼100 potency and 4-fold greater effectiveness than **(1)** upon intraperitoneal (ip) administration, demonstrating PAM activity *in vitro* and *in vivo* (Mishra et al., 1997). **(43)** showed *in vivo* activity at reducing social and cognitive schizophrenia-like symptoms in some but not all preclinical rodent models (Dyck et al., 2011; Beyaert et al., 2013; Daya et al., 2018). Though the precise mechanism is unclear, as clinical D_2_ antagonists reduce schizophrenia symptoms, the authors report PAOPA normalizes dopamine levels in their models, perhaps due to the availability of D_2_ autoreceptors.

### β_2_AR Allosteric Modulators: Hits From a DNA-Encoded Library With Peptidomimetic Features

**FIGURE 10:**
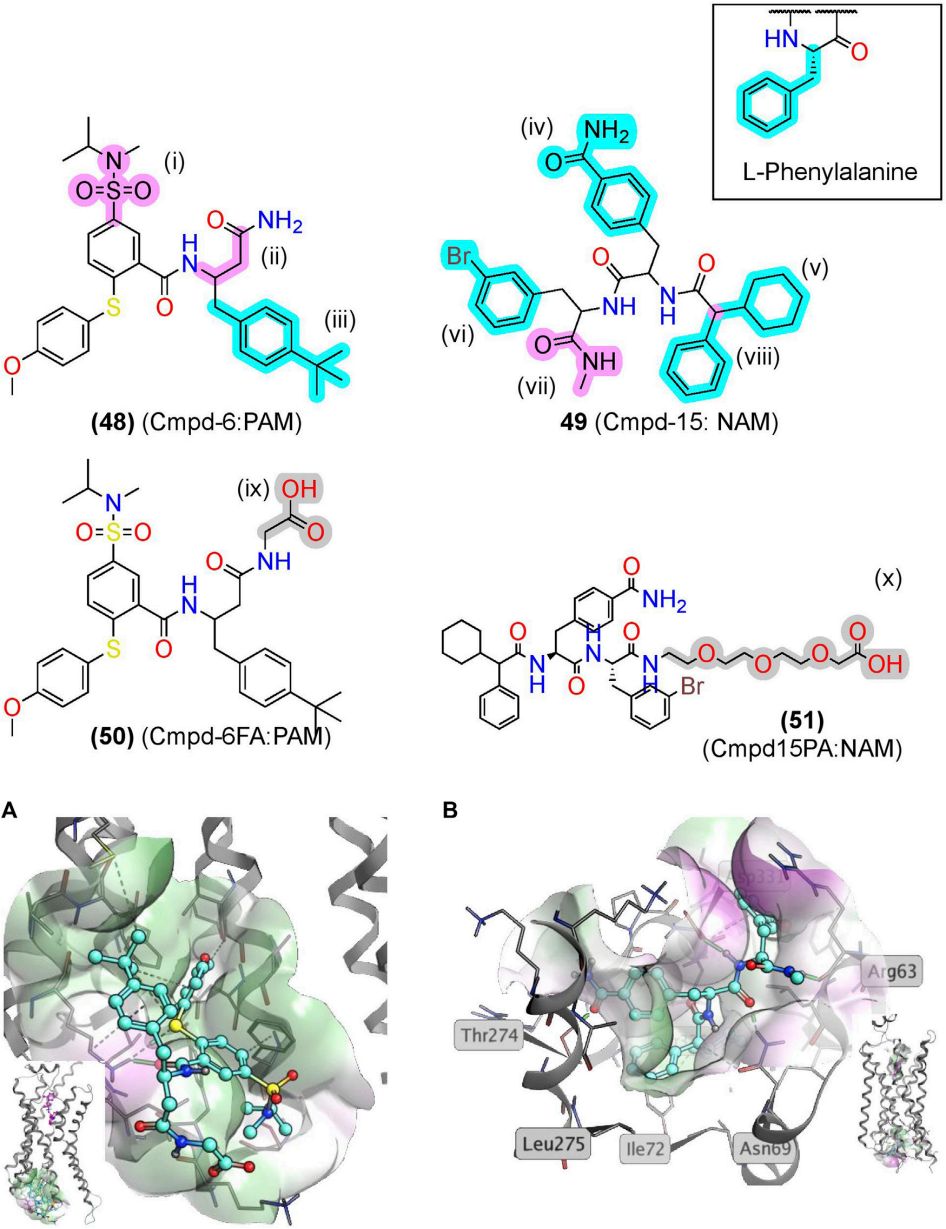
βAR2 AMs identified from exogenous synthetic libraries are polyamides with peptidomimetic-like features. **(A)** The β2AR cocrystallized with the PAM, Cmpd-6, bound to an intracellular binding pocket [PDB Code: 6N48 (Liu et al., 2019)]. (*Inset*) Side view with orthosteric ligand shown in pink. **(B)** The β2AR cocrystallized with the NAM, Cmpd-15, bound at an intracellular [PDB Code: 5X7D (Liu et al., 2017)]. (*Inset*) Side view with orthosteric ligand was shown in pink. Amide isosteres and backbone modifications are highlighted in pink; sidechain isosteres were highlighted in cyan. Van der Waals surface was shown in semitransparent surface (green = lipophilic; purple = hydrophilic). The in-text compound number is bold, followed by original name: activity. Structures were originally reported by Ahn et al., 2017 and Ahn et al., 2018. Figure was created with Chemdraw20.0 Marvin Sketch^5^ and MOE.^2^

Screening a DNA-encoded library (DEL) at ß_2_AR identified two small-molecule peptide/peptidomimetics—a PAM, Cmpd-6 **(48)**, and a NAM, Cmpd-15 **(49)** (Figure 10) (Ahn et al., 2017; Ahn et al., 2018). While **(48)** and **(49)** are not traditional peptidomimetics designed from endogenous peptides, we include them here as they contain multiple amide bonds and exemplify several peptidomimetic features. Both contain several amide bonds, amide bioisosteres, and other Cα backbone modifications, including a sulfonamide (i), a β-amino acid (ii), an N-methyl formamide (vii), and removal of the amino terminus (viii) (Figures 6, 10, pink). Moreover, **(48)** and **(49)** contain several sidechain bioisosteres of the endogenous F residue (Figure 10 inset, iii, iv, v, and vi) (Ahn et al., 2018). Moiety (v) consists of a α-phenylglycine and an α-cyclohexyl, which constrains the χ angles and appears necessary for binding in the R_I_ ß_2_AR structure cocrystallized with compound **(51)**, an analog of **(49)** (Figure 10B). Similarly, a “natural” peptide typically contains an NH_3_^+^ at (viii), which would likely reduce affinity by burying a positive charge in a hydrophobic pocket (Figure 10B). In the PAM R_A_ crystal structure, **(50)** produces numerous hydrophobic contacts with the intracellular binding pocket, forming an H-bond between the K149^4.41^ and carbonyl backbone (Figure 10A) (Liu et al., 2019).

Notably, nearly all the “peptidomimetic modifications” in **(48)** and **(49)** increase hydrophobicity and reduce flexibility compared to endogenous peptides (Figure 10), which are general trends for AMs as a drug class (Liu et al., 2020). Therefore, peptidomimetic modifications that reduce flexibility and increase hydrophobicity may improve AM peptidomimetic design, as peptides typically begin more hydrophilic and flexible than small molecules. Further investigation of peptidomimetic AMs should determine the utility of the hydrophobic and rigid constraints to see if these relationships hold for peptidomimetic AMs.

### Conjugating Functional Moieties to AMs to Improve Bioavailability and Physicochemical Properties

#### PAR1 Pepducin Biased Allosteric Modulators: PZ-128 Development, Entry into Clinical Trials, and Strategies to Further Improve Peptide/Peptidomimetic Bioavailability

**FIGURE 11:**
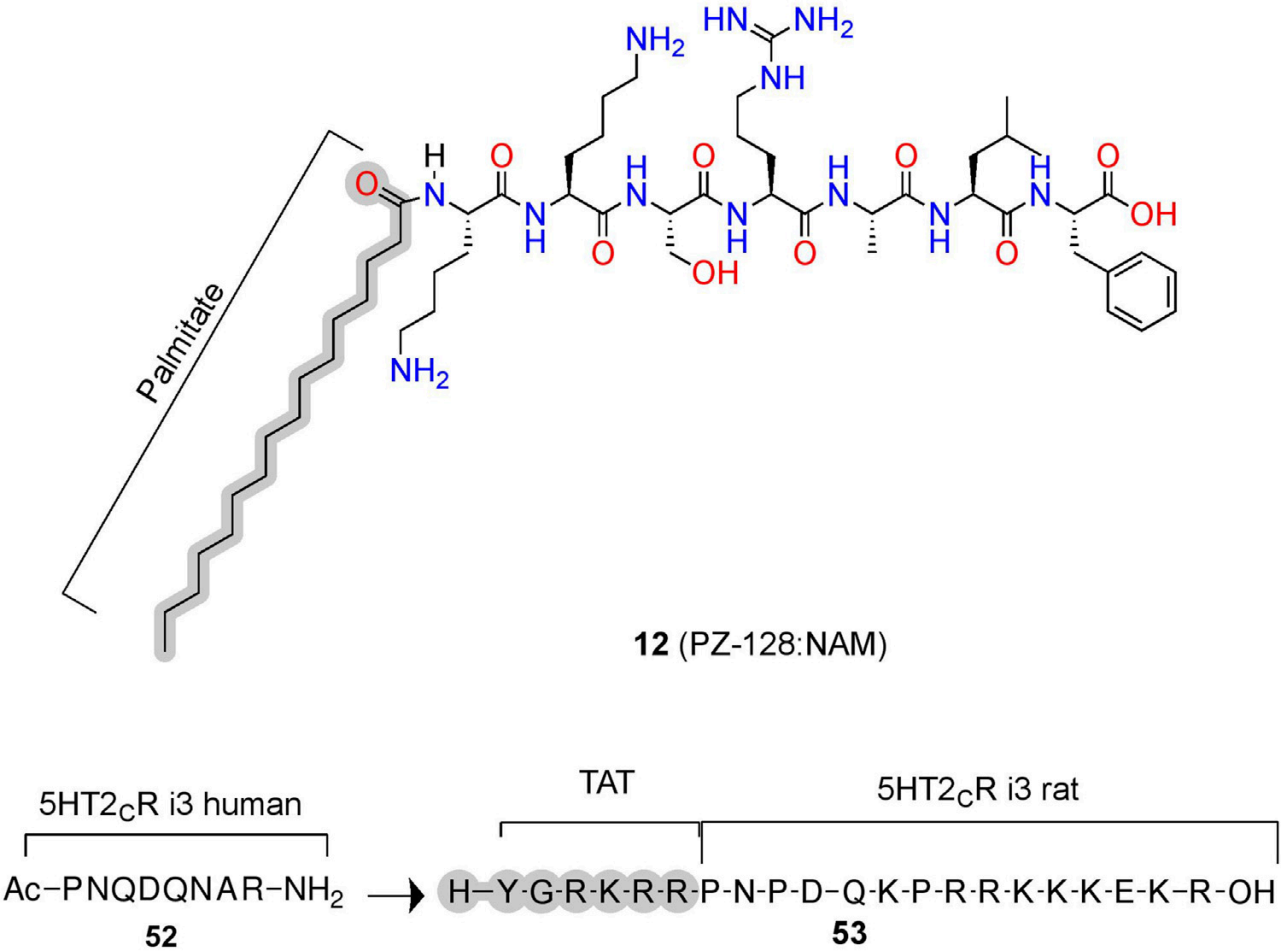
Peptide conjugations to improve physicochemical properties of pepducins at PAR1 and 5-HT_2C_. Example of pepducins derived for the ICL sequences that are unconjugated (acetyl) or conjugated to a palmitate- or cell-penetrating sequence (e.g., TAT). The in-text compound number is bold, followed by original name: activity. Structures were originally reported by Ji et al., 2006 and Anastasio et al., 2013. Figure was created with Chemdraw20.0, MarvinSketch,^5^ and Molecular Operating Environment (MOE).^2^

PZ-128 **(12)** is an AM derived from ICL3 of the PAR1 currently in clinical trials to treat coronary artery disease (Figure 11; Table 2) (Covic et al., 2002; Gurbel et al., 2016). **(12)** selectively inhibits PAR1 thrombin-mediated signaling, platelet aggregation, and thrombosis but not PAR4 thrombin-mediated effects, whose blockade leads to bleeding and coagulation problems (Trivedi et al., 2009; Zhang et al., 2012). Structurally, NMR studies of **(12)** show its conformation closely resembles the predicted R_i_ α-helical conformation of PAR1 ICL3, consistent with a NAM stabilizing the inactive receptor (Zhang et al., 2012). **(12)** is another prime example of the highly desirable and specific biological specificity achievable with peptidomimetic AMs, which helped facilitate its entry to clinical trials.

In phase II trials, patients tolerated PZ-128 **(12)** well, and an exploratory endpoint measuring major adverse coronary events and myocardial injury showed fewer events in the PZ128 group than in the placebo group (Kuliopulos et al., 2020). Impressively, the palmitate conjugation to an otherwise unmodified heptapeptide produced the bioavailability, metabolic stability, and membrane permeability necessary for *intravenous* administration in clinical trials of an intracellular target (Figures 3C, 11, gray). If *intravenous* administration is suboptimal, peptidomimetic strategies to improve oral availability, blood-brain barrier permeability, and general bioavailability include formulations and chemical modifications such as glycosylation, N-methylation, and more [e.g., Vagner et al. (2008), Moradi et al. (2016), Qvit et al. (2017), Lenci and Trabocchi (2020), Zizzari et al. (2021)].

#### 5-HT_2C_ AMs: Conjugations to Cell-Penetrating Peptides Increase Membrane Permeability

Similar to lipidation, the conjugation of cell-penetrating peptides can improve bioavailability (Figures 3C, 11). For example, **(52)** is a PAM at the human 5-HT_2C_ derived from ICL3, which increases the orthosteric agonist’s efficacy, as measured via Ca_i_^2+^ assays (Figure 11) (Ji et al., 2006; Anastasio et al., 2013). To improve bioavailability for *in vivo* studies, researchers added a short cell cell-penetrating peptide (Y-G-R-K-R-R)—called a transactivator of transcription (TAT)—to the homologous 5-HT_2C_ ICL3 rat sequence generating **(53)** (Ji et al., 2006; Anastasio et al., 2013). Conjugation with a similar cell-penetrating sequence to the human homolog produced **(19)**, leading to improved bioavailability, facilitating membrane insertion, and providing access to the receptor’s cytosolic face in human model systems (Brooks et al., 2005). **(52)** disrupts the complex between 5-HT_2C_ and PTEN—a lipid phosphatase that reduces 5-HT_2C_ signaling *in vitro* and *in vivo* (Ji et al., 2006; Anastasio et al., 2013). Critically, the PTEN:5-HT_2C_ complex provides a useful target for 5-HT_2C_ selectivity over 5-HT_2A_, as PTEN does not recognize 5-HT_2A_. However, the mechanism and binding site of **(52)** is unclear, as molecular modeling predicts these pepducins may bind the PTEN domain instead of the receptor, which would mean they act as PTEN:5-HT_2C_ protein-protein interaction inhibitors instead of classical AMs (Anastasio et al., 2013). Regardless of their mechanisms, these compounds produce *in vitro* and *in vivo* PAM activity at 5-HT_2C_, whether via targeting the receptor directly or disrupting a protein interaction that acts as a NAM. Either strategy can prove useful if there is a known protein allosteric modulator and blocking protein-protein interactions provide a compelling alternative strategy to develop AMs indirectly.

#### ß_2_AR Allosteric Modulators: Conjugations to Hydrophilic Moieties to Improve Solubility

In addition to increasing membrane permeability, conjugations can improve a compound’s physicochemical properties. To facilitate crystallization of ß_2_AR with a bound AM, researchers designed derivatives of Cmpd-6 **(48)** and Cmpd-15 **(49)** to increase their solubility, enabling higher AM concentrations during the crystallization process. Attaching a polyethylene glycol- (PEG) to **(49)** led to Cmpd-15PA **(51)** (referred to as Cmpd15PA), which facilitated the purification of a NAM ß_2_AR R_i_ crystal structural (Liu et al., 2017). Analogously, attachment of glycine (G) with a COO^−^ to **(48)** led to Cmpd-6FA **(50),** enabling the PAM ß_2_AR R_A_ crystal structure (Liu et al., 2019). Peptidomimetic’s modifiable N-termini and C-termini make conjugation of functional moieties to improve their biological or physiochemical properties (Figure 6) easy and predictable.

### Global Restrictions to Stabilize Peptide Conformation or Improve Druggability

Global restrictions are macrocyclizations made by forming a covalent bond between two nonsequential peptide residues, such as 1) the N-termini to the C-termini (head:tail), N-termini to a side chain (head:side chain), a side chain to a side chain (sidechain: sidechain), or side chain to carboxy-terminus (side chain:tail) (Figure 5C). Macrocyclizations take forms, including lactams, lactones, peptides, “stapled peptides,” ring-closing metathesis, azide-alkyne cycloadditions, disulfides, biaryl cross-linkages, and more. Here, we focus on macrocyclizations’ impact on pharmacodynamic and pharmacokinetic parameters; for details on their synthetic schemes, see reviews by White and Yudin (2011) and Reguera and Rivera (2019). Global restrictions often stabilize bioactive secondary structures such as β-loops, β-hairpins (Robinson, 2008), β-strands (Loughlin et al., 2004; Tanaka et al., 2020), and α-helices (Garner and Harding, 2007) to improve their activity while also enhancing their metabolic stability and bioavailability.

#### ß_2_AR Allosteric Modulators: Sidechain:sidechain Peptide Stapling of *i* and *i*+4 to Stabilize α-helix Conformations Derived From Gα_s_

**FIGURE 12:**
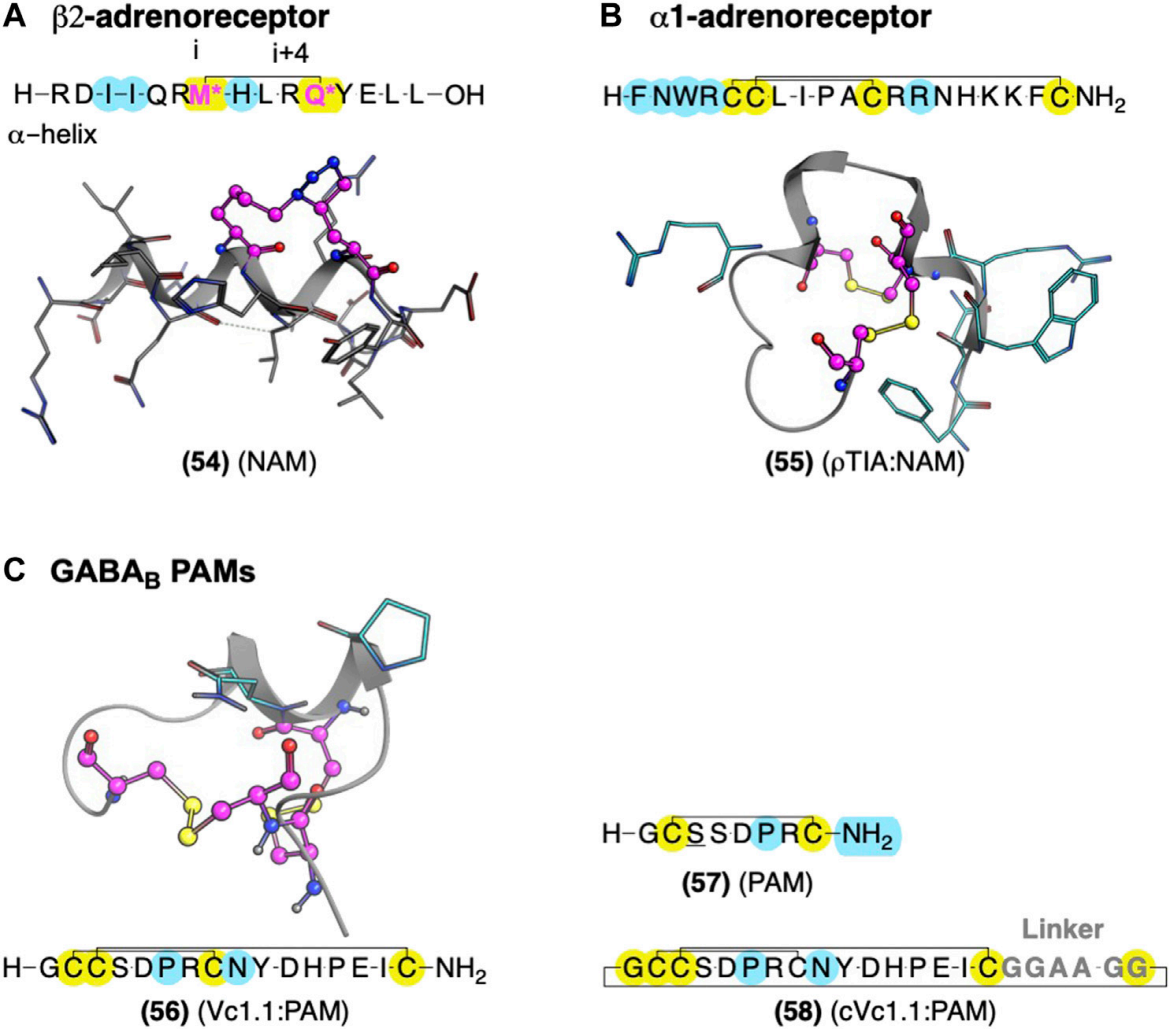
Global conformational restraints to stabilize secondary structures with AMs identified from Gα_s_ and conotoxins. **(A)** Model of stapled peptides from Gα_s_ to stabilize αhelix based on the crystal structure (PDB Code: 3SN6) (Rasmussen et al., 2011). **(B)** Conotoxin AMs ρTIA at the α1-adrenoreceptor and α-conotoxin Vc1.1 at the GABA_B_ stabilized by disulfide bonds—NMR structures ρTIA NMR (PDB Code: 2LR9) (Ragnarsson et al., 2013) and **(C)** NMR structure of Vc1.1 (PDB Code: 2H8S) (Clark et al., 2006). Yellow highlights residues involved in macrocyclization. * (pink letters) indicate side chains replaced with an alkyne and azide group to mediate “stapling.” Yellow indicates residues involved in macrocyclization. Key residues for bioactivity are shown in cyan. The in-text compound number is bold, followed by original name: activity. Structures were originally reported by Krajewski et al., 2001; Sharpe et al., 2003; and Boyhus et al., 2018. Figure was created with Chemdraw20.0, MarvinSketch,^5^ and Molecular Operating Environment (MOE).^2^

Several crystal structures of GPCRs bound to their Gα exist, which provide compelling leads for AM development, as Gα acts as protein PAMs. Boyhus et al. (2018) used the ß_2_AR-Gα_s_ crystal structure (PDB ID: 3SN6) to design **(54)**, a NAM derived from the 15-amino acid sequence covering the Gα_s_ α-helix which contacts the receptor (Figure 12). They stabilized the α-helix with the well-established ‘peptide stapling’ technique, which covalently links a sidechain alkyne and azide at the *i* and *i+4* positions, forming a 1,2,3-triazole through a copper-catalyzed cycloaddition. Several peptides showed NAM activity, reducing the maximal efficacy of cAMP formation by the agonist isoproterenol and modestly decreasing radiolabeled agonist affinity (Boyhus et al., 2018). Unfortunately, these NAMs are not likely to show high selectivity for β_2_AR over other Gα_s_ receptors. However, the available crystal structures could facilitate ligand design to interact with receptor-specific residues to enhance selectivity by identifying nonconserved residues near the Gα contacts.

#### M_1_AChR and α1_B_AR Allosteric Modulators: Conotoxins With Compact Secondary Structure Motifs Stabilized by Disulfide Bridges

Peptides from nature-derived sources have yielded AMs at the gamma-aminobutyric acid B receptor (GABA_B_) (Daniel and Clark, 2017), M_1_AChR (Krajewski et al., 2001; Mourier et al., 2003), and α_1B_AR (Sharpe et al., 2003). These leads contain multiple cysteine disulfide bonds that stabilize compact loops and structural motifs considered “privileged scaffolds” with broad applicability in drug development (Figure 12) (Jin et al., 2019). One highly desirable feature of these nature-derived peptide leads is that they cover chemical-space that is not easily accessible in synthetic small molecule libraries (Muratspahić et al., 2019).

One example is ρ-TIA **(55)** – a 19 amino acid conopeptide with disulfides between cysteines (Cys) Cys5-Cys11 and Cys6-Cys19 – that binds to the α1BAR ECLs, producing NAM activity (Sharpe et al., 2001; Sharpe et al., 2003; Ragnarsson et al., 2013). An alanine scan showed the importance of the 3rd position tryptophan (W), the 4th position R, the 7th position L, the 8th position isoleucine (I), and a modest effect of 12th position R (Figure 12B, *cyan*) (Sharpe et al., 2003). Furthermore, Ragnarsson et al. (2013) identified the key pharmacophore contacts between ρ-TIA and α_-1B_AR using homology models, molecular docking, functional mutagenesis studies, and NMR studies (Ragnarsson et al., 2013). These studies expanded on the alanine scan results, showing a salt-bridge between R4 of ρ−TIA and the receptor’s D327; additionally, these studies proposed a π−π interaction between ρTIA W3 and the receptor F330. Thus, ρ-TIA binding to the α-_1B_AR ECLs provides an excellent example of using biophysical methods to inform the design and guide the future AM development at the vestibule site.

Another nature-derived source of peptide AMs includes three-finger proteins (3FP) which are a family of venom proteins with three variable loops (or fingers) connected to a globular domain with multiple disulfide bonds; the three “fingers” provide a scaffold to generate selective high-affinity interactions with receptor ECLs (Marquer et al., 2011; Servent et al., 2011; Fruchart-Gaillard et al., 2012). One such protein, muscarinic toxin-7 (MT7), isolated from mamba snake venom, is an M_1_AChR NAM with greater >10,000-fold selectivity over M_2_AChR, more than any other M_1_AChR ligand known (Maeda et al., 2020). MT7 produces its NAM activity by increasing the on-rate of antagonists and increases agonists’ off-rate to the receptor (Olianas et al., 2004), with “finger 2” sterically blocking access to the orthosteric site (Maeda et al., 2020). Using the MT7 scaffold, Maeda et al. (2020) used a phage display library and the known MT7 and M_1_AChR contacts to identify the Tx24 mutant—a NAM selective for M_2_AChR over M_1_AChR (Maeda et al., 2020). Thus, directed evolution of 3FPs and related nature-derived scaffolds allows for identifying new AM lead sequences.

Limitations of nature-derived peptides include their large size and labile disulfides susceptible to reduction, thiol exchange, and enzymatic cleavage. However, numerous sidechain:sidechain amino acid pairs are available to replace the metabolically labile disulfide (Kennedy et al., 2020). Further work is required to minimize the distance between the key pharmacophore features of most nature-derived peptides, such as restraints and sidechain isosteres of “finger 2” in the MT7 example, which form most of the receptor contacts. Decreasing the size and excess amino acid residues can improve the druggability and synthetic feasibility of such scaffolds.

#### GABA_B_ Allosteric Modulator: Vc1.1 Conotoxin and Head:Tail Cyclization Produce an Orally Available Peptidomimetic

The α-conotoxin Vc1.1 **(56)** is a 16-residue peptide with disulfide bonds between Cys2-Cys8 and Cys3-Cys16 derived from marine cone snails (Figure 12C, yellow) (Clark et al., 2006). **(56)** indirectly blocks N-type Ca_v_ channels by targeting GABA_B_ and nicotinic acetylcholine receptors (nAChRs), producing analgesia in animal models (Clark et al., 2006). Accordingly, **(56)** entered clinical trials for neuropathic pain. Unfortunately, trials were discontinued because the α9α10 nicotinic acetylcholine receptors (nAChRs) activity was thought to contribute to its analgesia in preclinical rodents, which does not translate well to humans, unlike the initially proposed nAChRs.^3,^ ^4^ However, more recent studies suggest its PAM activity at GABA_B_ is sufficient for its Ca_v_2.2 inhibition and thus its *in vivo* antinociception (Callaghan et al., 2008; Sadeghi et al., 2017), rekindling interest in Vc1.1 as a potential lead for safer analgesics for the treatment of pain.

The low selectivity of Vc1.1 **(56)** for GABA_B_ over α9α10 nAChRs led to several studies determining the receptor binding sites and Vc1.1 pharmacophore responsible for its affinity and activity at each receptor. Mutagenesis studies at GABA_B1_ indicated Vc1.1 does not bind to the orthosteric site, and its activity requires the obligatory GABA_B2_ receptor, containing an allosteric site (Huynh et al., 2015). Computational modeling suggests Vc1.1 binds to the heterodimer interface of the GABA_B1_ and GABA_B2_ (Adams and Berecki, 2013). Comparison of **(56)** to structurally and functionally related conotoxins, including Tx1.2, Kn1.2, Bu1.1, Ai1.2, Pn1.2, and Pu1.2, identified **(57)** as the minimum GABA_B_ PAM pharmacophore, again measured via inhibition of calcium channel currents. **(57)** includes residues 1-8 and a single disulfide bond (Figure 12C) (Carstens et al., 2016). These results match alanine-scans showing substitutions at D11, glutamic acid (E)15, and I15 did not shift the AMs potency (Sadeghi et al., 2018). While truncations showed position 9 is not essential for GABA_B_ activity (Carstens et al., 2016), it does contribute to selectivity between GABA_B_ and α9α10 nAChRs (Figure 12C, cyan). Starting with **(56)**, the substitution of asparagine (N) 9→R (Vc1.1[N9R]) drastically increased selectivity for GABA_B_ over α9α10 (Cai et al., 2018). Consistent with a GABA_B_ analgesic mechanism, these more selective GABA_B_ PAMs produced similar (or better) analgesia in animal models than the parent compound Vc1.1.

Despite the many desirable drug-like characteristics of nature-derived peptides, such as **(56)**, they are not typically orally bioavailable—requiring subcutaneous or intramuscular injection—and suffer from poor metabolic stability. To address these problems, Clark et al., 2010 cyclized an analog of **(56)** at the C-terminus and N-terminus, a head:tail cyclization (Figure 12C, yellow). Their design included an inert C-terminal “linker sequence” -G-G-A-A-G-G- to reduce undesired conformational constraints and minimize ‘disulfide shuffling’ in which rearrangement of the disulfide bonds under biological conditions that lead to inactive isomers (Figure 12, gray). Impressively, the resultant compound, cVc1.1 **(58)**, is orally available in preclinical analgesia models (Clark et al., 2010). To further improve stability and eliminate potential “disulfide shuffling” that produces inactive isomers, Yu et al., (2015) used NMR and computational modeling to design smaller ligands with one disulfide bond instead of two. The stabilization of the bioactive α-helix of **(58)** led to more metabolically stable compounds with only modest decreases in potency. These chemical modifications provide a few of the many techniques used to further increase nature-derived peptides’ drug-like properties (Norton, 2017).

#### Urotensin II Receptor Allosteric Modulators: Converting Orthosteric Ligands to AMs Using Scaffold Replacement

**FIGURE 13:**
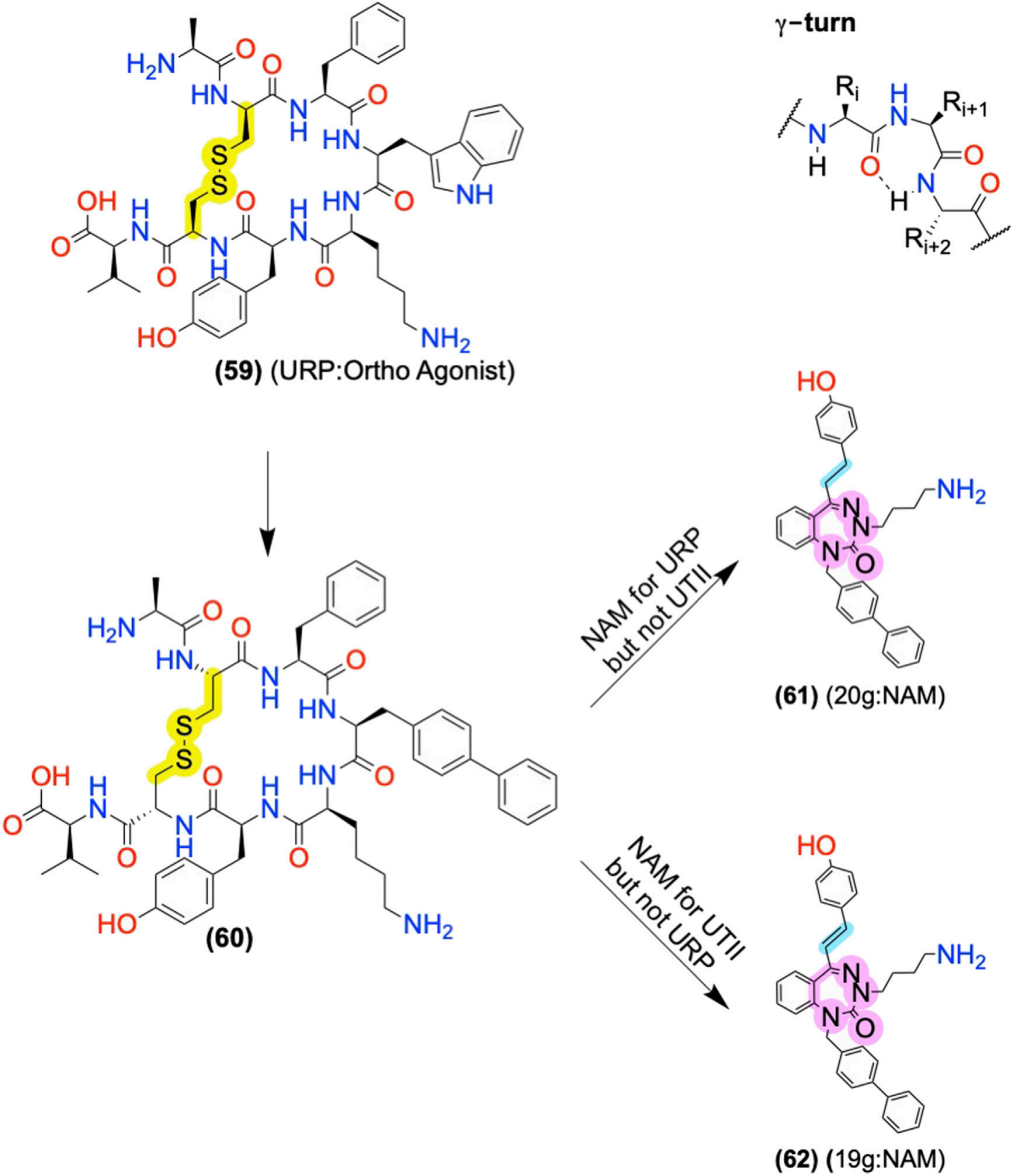
Global restraints and scaffold replacement to identify probe-dependent AMs at UTII. The disulfide bond stabilizes the γ-turn. Substitution of peptide backbone that retained key pharmacophore elements led to probe-dependent NAMs for either endogenous orthosteric agonist, URP or UTII. Cyan highlights “sidechain” differences between **(61)** and **(62)**. The yellow highlights show a structural difference that leads to probe dependence. The in-text compound number is bold, followed by original name: activity. Structures were originally reported by Chatenet et al., 2013; Dufour-Gallant et al., 2015; and Douchez et al., 2017. Figure was created with Chemdraw20.0.

At the Urotensin II Receptor (UTS2R), the two endogenous orthosteric agonists, URP and urotensin II (UII), display different biased signaling profiles that may contribute to heart failure in distinct ways (Billard et al., 2019) with URP concentration being 10× higher than UTII in plasma of patients with acute heart failure (Jani et al., 2013). Modifications to URP converted it from an orthosteric agonist to a probe-dependent allosteric modulator. Amino acid substitutions to URP **(59)** produced a UTS2R NAM **(60)**, with probe-dependent selectivity for URP and UII (Figure 13) (Chatenet et al., 2012). A scaffold replacement with a 1,3,4-benzotriazepin-2-one produced **(61)** and **(62),** which mimicked the γ-turn stabilized by the disulfides, and also produced probe-dependent NAMs (Chatenet et al., 2013; Dufour-Gallant et al., 2015; Douchez et al., 2017). **(61)** is a NAM for the endogenous URP agonist but showed minimal NAM activity for the UII in an *ex vivo* rat aortic ring vasoconstriction assay. Alternatively, **(62)** is a NAM for UII but not URP (Douchez et al., 2017) (Figure 13). While several analogs displayed probe-dependent selectivity, the subtle differences between **(61)** and **(62)**—with a switch from an alkane to an alkene—show that even minor changes can switch probe dependence (Figure 13, yellow). Since the parent compounds are orthosteric agonists, future work in the UTS2R system includes identifying the allosteric site. Two possible mechanisms could explain these allosteric results: 1) the orthosteric agonists act as bitopic ligands, with an allosteric site in the receptor vestibule, or 2) the new “AMs” change the binding cooperativity of endogenous ligands by binding to the orthosteric site of an oligomer.

Nonetheless, the ability to create a probe-dependent AM for one endogenous ligand, but not another, is a compelling tool to hone a drug’s biological specificity or use as a pharmacological tool to better understand the role of individual receptor/ligand pairs. Further studies will have to determine whether this probe-dependent selectivity is therapeutically and physiologically relevant at the UTS2R and other GPCRs.

## Conclusion and Future Directions

Peptides and proteins are a bountiful source for identifying AM leads, including those derived from 1) endogenous AM proteins and protein fragments, 2) endogenous bitopic ligands, 3) intramolecular contacts (e.g., pepducins or ECL contacts), 4) endogenous protein-protein interactions (transducer proteins, accessory proteins, scaffold proteins, and antibodies), 5) nature-derived libraries (e.g., conotoxins and cyclotides), 6) synthetic exogenous libraries (e.g., combinatorial or DNA encoded libraries), and 7) directed evolution (e.g., phage display) (Figure 2). AM leads from these sources can offer distinct advantages over small molecule HTS hits, including having an established putative binding site with rich structural and pharmacophore information. Additionally, lead identification may require synthesis and testing of relatively few compounds with access to distinct chemical space (Muratspahić et al., 2019), enabling them to bind better to the relatively shallow cavity of most allosteric sites (Ivetac and McCammon, 2010; Chang et al., 2013; Leshchiner et al., 2015). Moreover, peptide/peptidomimetic AM leads are synthetically accessible, often with high affinity, efficacy, and tolerability; emerging studies demonstrate their capability to produce extraordinary biological specificity through biased or probe-dependent AMs.

Furthermore, peptide drugs’ traditional limitations—poor metabolic stability and low bioavailability—are increasingly surmountable, with multiple peptidomimetic AMs reaching clinical trials, including PZ-128 (Kuliopulos et al., 2020) at PAR1 and Vc1.1 at GABA_B_^3^. Even if the pharmacokinetic issues prove unsurmountable, peptide AM leads can serve as pharmacological tools to help validate (or invalidate) targets *in vivo*. When the peptide pharmacophore is small and well-defined, conversion to a small molecule is relatively straightforward for “small-molecule like peptidomimetics.” In cases containing long lead sequences, with pharmacophore features separated by large distances, peptidomimetic conversion to a small molecule may not be possible; in these cases, utilizing “peptide-like peptidomimetic” strategies is optimal (Figure 3). Notably, modifications to reduce flexibility and increase hydrophobicity may prove more important for AMs than other targets as AMs are generally more lipophilic with fewer rotatable bonds than drugs at other target classes (Liu et al., 2020).

Future peptide and peptidomimetic AM drug discovery efforts face several exciting prospects and challenges, including an improved ability to convert peptide pharmacophores into small molecules. Peptidomimetic AMs can harness structures already refined through millions of years of evolution combined with the pharmacokinetic practicality of small molecules. Relatedly, employing directed evolution from leads to perform SAR is particularly exciting as libraries can be fine-tuned based on biological readouts. While peptides (and proteins) are increasingly druggable, optimizing their drug-like properties is still not trivial. However, the application and further development of peptide formulations provide promise on this front. Moreover, combining the benefits of AMs with advantages from other drug design principles, such as targeting multiple receptors to reduce side effects and improve effectiveness, is a promising future direction for the field (Giri et al., 2015; Olson et al., 2017; Olson et al., 2019; Hillhouse and et al., 2021). Lastly, peptide and peptidomimetic AMs designed from protein-protein interactions that harness structure-based drug design principles are promising future avenues with the increasing availability of GPCR:protein structures. While small molecules should and will continue to play a role, peptides offer a complementary, often underappreciated, and mostly untapped source for AM lead identification and drug discovery.

## Footnotes

1
BioRender.com.

2 (2019.01) Molecular Operating Environment (MOE), in, Chemical Computing Group Montreal, QC, Canada.

3
https://www.asx.com.au/asxpdf/20061123/pdf/3zqm91n1jhpff.pdf.

4
https://www.asx.com.au/asxpdf/20070814/pdf/313yjgpf7jl4lg.pdf.

5 MarvinSketch was used to draw and display MOE’s chemical structures; MarvinSketch 18.10.0, 2018, ChemAxon (http://www.chemaxon.com).

## Glossary

3FP: Three-finger protein
5HTR: Serotonin receptor
α_1B_AR: α_1B_ adrenergic receptor
A: Alanine
AC: Adenylyl cyclase
Ago-PAMs: Ago-positive allosteric modulators
AM: Allosteric modulator
AT_2_R: Angiotensin II receptor
ß_2_AR: ß_2_-Adrenergic receptor
C or Cys: Cysteine
CB_1_: Cannabinoid 1 receptor
CB_2_: Cannabinoid 2 receptor
CPP: Cell-penetrating peptide
CDRs: Complementary determining regions
CXCR4: C-X-C chemokine receptor type 4
D: Aspartic acid
D_2_: Dopamine 2 receptor
DEL: DNA-encoded libraries
DOR: Delta opioid receptor
E: Glutamic acid
ERK1/2: Extracellular signal-regulated kinases
ECL: Extracellular loop
hFSHR: Human follicle stimulating hormone receptor
ICL: Intracellular loops
EP4: Prostaglandin E_2_ receptor 4
F: Phenylalanine
FSH: Follicle stimulating hormone
FP: Prostaglandin F receptor
FPR3: Formyl peptide receptor 3
GHSR: Growth hormone secretagogue receptor
GHRP: Growth hormone-releasing peptide
H: Histidine
JNK: c-jun N-terminal kinase
GPCR: G-protein coupled receptor
GRKs: G-protein coupled receptor kinases 2/3
GABA_B_: Gamma-aminobutyric acid B receptors
GDP: Guanosine diphosphate
GLP1: Glucagon-like peptide-1
GTP: Guanosine triphosphate
HTS: High-throughput screening
I: Isoleucine
K: Lysine
L: Leucine
M: Methionine
M_2_: Muscarinic 2 receptors
MOR: mu-opioid receptor
N: Asparagine
NMR: Nuclear magnetic resonance
NAM: Negative allosteric modulator
NAL: Neutral allosteric ligands
OTR: Oxytocin receptor
P: Proline
PAM: Positive allosteric modulator
PEG: Polyethylene glycol
PGF2α: Prostaglandin F2α
PAR1: Protease-activated receptor 1
ProtCID: Protein common interface database
PTEN: Protein phosphatase and tensin homolog
R: Arginine
RAMPs: Receptor activity modulating proteins
RCPs: Receptor complement proteins
RTKs: Receptor tyrosine kinases
S: Serine
SAR: Structure-activity relationships
T: Threonine
TAT: Transactivator of transcription
TM: Transmembrane
URP: Urotensin-related peptide
UT-II: Urotensin II
UTS2R: Urotensin II receptor
V: Valine
V_2_R: Vasopressin 2 receptor
W: Tryptophan
Y: Tyrosine
